# Ablation of the Regulatory IE1 Protein of Murine Cytomegalovirus Alters *In Vivo* Pro-inflammatory TNF-alpha Production during Acute Infection

**DOI:** 10.1371/journal.ppat.1002901

**Published:** 2012-08-30

**Authors:** Sara Rodríguez-Martín, Kai Alexander Kropp, Vanessa Wilhelmi, Vanda Juranic Lisnic, Wei Yuan Hsieh, Mathieu Blanc, Andrew Livingston, Andreas Busche, Hille Tekotte, Martin Messerle, Manfred Auer, Iain Fraser, Stipan Jonjic, Ana Angulo, Matthias J. Reddehase, Peter Ghazal

**Affiliations:** 1 Division of Pathway Medicine and Centre for Infectious Diseases, University of Edinburgh, Edinburgh, United Kingdom; 2 Institute for Virology, University Medical Center of the Johannes Gutenberg University, Mainz, Germany; 3 Department of Histology and Embryology/Center for Proteomics, Faculty of Medicine, University of Rijeka, Rijeka, Croatia; 4 Department of Virology, Hannover Medical School, Hannover, Germany; 5 Wellcome Trust Centre for Cell Biology, University of Edinburgh, Edinburgh, United Kingdom; 6 University of Edinburgh, School of Biological Sciences (CSE) and School of Biomedical Sciences (CMVM), Edinburgh, United Kingdom; 7 Laboratory of Systems Biology, National Institution of Allergy and Infectious Disease, National Institutes of Health, Bethesda, Maryland, United States of America; 8 Institut d'Investigacions Biomediques August Pi i Sunyer, Barcelona, Spain; University of Alabama at Birmingham, United States of America

## Abstract

Little is known about the role of viral genes in modulating host cytokine responses. Here we report a new functional role of the viral encoded IE1 protein of the murine cytomegalovirus in sculpting the inflammatory response in an acute infection. In time course experiments of infected primary macrophages (MΦs) measuring cytokine production levels, genetic ablation of the immediate-early 1 (*ie1*) gene results in a significant increase in TNFα production. Intracellular staining for cytokine production and viral early gene expression shows that TNFα production is highly associated with the productively infected MΦ population of cells. The *ie1-* dependent phenotype of enhanced MΦ TNFα production occurs at both protein and RNA levels. Noticeably, we show in a series of *in vivo* infection experiments that in multiple organs the presence of *ie1* potently inhibits the pro-inflammatory cytokine response. From these experiments, levels of TNFα, and to a lesser extent IFNβ, but not the anti-inflammatory cytokine IL10, are moderated in the presence of *ie1*. The *ie1-* mediated inhibition of TNFα production has a similar quantitative phenotype profile in infection of susceptible (BALB/c) and resistant (C57BL/6) mouse strains as well as in a severe immuno-ablative model of infection. *In vitro* experiments with infected macrophages reveal that deletion of *ie1* results in increased sensitivity of viral replication to TNFα inhibition. However, *in vivo* infection studies show that genetic ablation of TNFα or TNFRp55 receptor is not sufficient to rescue the restricted replication phenotype of the *ie1* mutant virus. These results provide, for the first time, evidence for a role of IE1 as a regulator of the pro-inflammatory response and demonstrate a specific pathogen gene capable of moderating the host production of TNFα *in vivo*.

## Author Summary

The suppression of the production rather than the blockage of action of the potent inflammatory mediator TNFα is a particular hallmark of anti-TNFα mechanisms associated with microbial and parasitic infections. Whether this mode of counter-regulation is an important feature of infection by viruses is not clear. Also, it remains to be determined whether a specific pathogen gene in the context of an infection *in vivo* is capable of modulating levels of TNFα production. In this study we disclose a virus-mediated moderation of TNFα production, dependent on the *ie1* gene of murine cytomegalovirus (MCMV). The *ie1* gene product IE1 is a well-characterized nuclear protein capable of altering levels of host and viral gene expression although its biological role in the context of a natural infection is to date unknown. We provide evidence showing that *ie1* is associated with a moderated pro-inflammatory cytokine response, in particular with TNFα production. Further, we show that the viral moderation of this cytokine is not only readily apparent *in vitro* but also in the natural host. The identification of a viral gene responsible for this mode of regulation *in vivo* may have therapeutic potential in the future in both anti-viral and anti-inflammatory strategies.

## Introduction

The β-herpesvirus human cytomegalovirus (HCMV) is a species-specific virus and a clinically important pathogen that can establish both acute and latent infections. The murine counterpart (MCMV) provides a useful model for studying CMV natural infection in its natural host. CMV has a dsDNA genome that is sequentially expressed in a hierarchical cascade, immediate early (IE), early (E) and late (L) [1]. The MCMV IE1 protein has been implicated in the transcriptional activation of viral early genes in combination with the IE3 protein [2] as well as in the expression of cellular genes [3]–[5]. The IE1-induced activation of gene expression is not completely understood, although the ability of IE1 to interact with chromatin through histones [6], [7] might be one mode of action responsible for its transactivating functions.

The ability of MCMV IE1 protein to activate cellular gene expression has been documented for genes involved in immune signalling pathways, DNA metabolism and cell cycle control [3], [4], [8], [9]. Recently, a single point mutation in MCMV IE1 has been shown to disrupt its capacity of trans-activating cellular genes ribonucleotide reductase and thymidylate synthase, involved in nucleotide metabolism [10]. IE1 is also a potent disruptor of promyelocytic leukemia gene product (PML) oncogenic domains (PODs/ND10) [11], [12], which have been implicated in intrinsic cell immunity to infection [13]–[15]. *In vitro*, an IE1-deleted MCMV grows with the same efficiency as wild type MCMV in different cell types [11]. However, this mutant is severely attenuated in immune competent BALB/c mice as well as in SCID mice lacking the adaptive immune control, and shows a reduced doubling time in various organs of BALB/c mice after hematoablative treatment [10], [11]. On the basis of these studies we have previously speculated about a putative role of IE1 in interfering with some early intrinsic or innate immune mechanism [11], involving pro-inflammatory cytokine production or signalling. In this context, the homologous HCMV IE1 protein has recently been reported to counteract the type I interferon response by targeting principally STAT2 [16]. Moreover, in the case of HCMV, there are an increasing number of studies pointing to IE1 promoting a nuclear environment conducive for viral expression by modulating epigenetic regulation of the viral major immediate early promoter (MIEP). In these studies, ND10 associated-proteins, in particular hDaxx, have been shown to be repressors of the MIEP [17]. In this scenario, the dispersion of ND10s by the IE1 protein at early time of infection is thought to increase MIEP transcription efficiency indicating a potential role of ND10s as part of an antiviral defence mechanism inactivated by IE1 [12]. However, it is important to note that in the absence of the *ie1* gene HCMV, unlike MCMV or rat CMV [18], displays growth impairment under conditions of low multiplicity of infection (MOI) on primary fibroblasts [19], [20]. The growth phenotype of the HCMV *ie1*-deficient strains adds an additional level of complication that can be avoided by utilising a rodent CMV model. Overall, while the mode of action of IE1 has been extensively studied in different species of CMV, the functional relationship of this protein in the regulation of host-virus interaction pathways, especially in a more biologically relevant context such as in infection of MΦ or at a whole organism level, remains unknown to date.

Macrophages (MΦs) play a central role in CMV infection with regard to viral dissemination, replication and establishment of latency [21], [22]. At the host response level, MΦs constitute one of the principal effectors of innate immunity. Upon activation, MΦs produce a number of pro-inflammatory cytokines such as TNFα, IL1, IL6, IFNα/β and IL12, which are reported to be key mediators of the response against MCMV [23]–[25]. Of these pro-inflammatory cytokines, TNFα represents a key player in innate immunity. It is produced in response to a wide range of pathogens and is a hallmark of inflammatory diseases. One of the main activities of this pro-inflammatory cytokine is to provide protection against pathogen invasion. It is therefore not too surprising that there exist a number of pathogens which have developed several mechanisms to inhibit or modulate different stages of the TNFα action, ranging from the blockage of TNFα binding to the receptor to inhibition of specific TNFα-induced responses, such as gene expression or caspase activation [26]–[28]. To date, both human and mouse CMV have been reported to block TNFα-mediated gene activation by interfering with cell signalling and TNFα receptor expression [29]–[31]. Others have shown that HCMV inhibits TNFα-induced caspase-dependent apoptosis by encoding viral inhibitors [32]. It has also been shown that MCMV blocks caspase-independent apoptosis by direct binding and degradation of receptor-interacting protein RIP1 by the viral M45 protein [33], [34].

Despite the several known viral strategies to modulate TNFα-induced responses, there are only a few examples in the literature of viruses that interfere with TNFα production [35], [36]. In contrast, numerous studies have shown that bacteria and parasites almost exclusively interfere with TNFα by blocking its production, specifically targeting p38 MAPK, JNK and NF-κB signalling pathways involved in the activation of the cytokine production. For instance, *Yersinia* outer protein (Yop) J has been reported to bind to members of the MAPK family and IκB kinase β, and interfere with the MAPK and NF-κB signal transduction responsible for activating TNFα production (reviewed in [37]). In addition, *Yersinia pestis* was reported to also block TNFα production in MΦs by inhibition of MAPK activation by the antigenic proteins Low calcium response V (LcrV) and Yop B [38]. Also in monocytes/MΦs, *Salmonella* SptP protein reduces TNFα production by blocking the Raf/MAPK signalling pathway [39] and *Escherichia coli* K1 protein specifically targets NF-κB for inhibition of the pro-inflammatory response [40]. Whether any of the described cell-culture characterised viral or microbial pathogen-mediated suppression of TNFα production also occurs *in vivo* in an intact physiological system is not known.

We report *in vivo and in vitro* studies disclosing a previously unrecognized biological role of MCMV in moderating the production of pro-inflammatory cytokines, in particular TNFα involving an IE1 dependent mechanism, detectable at the protein and transcriptional level, in both immune intact strains of mice and in a severe immune-ablative model of infection. While the loss of *ie1* results in increased sensitivity of viral replication in macrophages to TNFα inhibition, *in vivo* the ablation of TNFα is insufficient to rescue the replication phenotype.

## Results

### A comparison of MCMV and MCMVdie1 infection in primary BMMΦ

**Figure 1:**
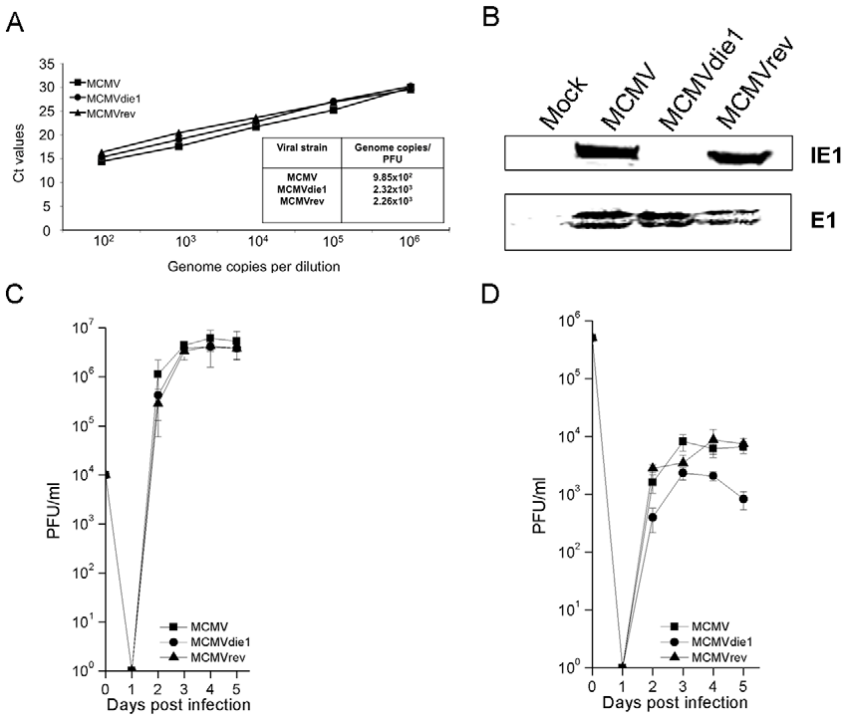
Comparison of MCMV, MCMVdie1 and MCMVrev. (A) Genome copy number-to-PFU ratios of MCMV, MCMVdie1 and MCMVrev stocks. Viral DNA was extracted from 200 µl of viral stock from each strain and the number of genome copies per ml was measured by qPCR and related to infectivity measured as PFU by virus plaque assay. (B) Western blot shows equal infection of BMMΦ. Total protein was extracted at 24 hpi and Western blot was carried out to assess the expression of both IE1 and E1 viral proteins, showing that cells were equally infected. (C) (D) Growth of MCMV, MCMVdie1 and MCMVrev in NIH-3T3 and BMMΦ cells. Cells were infected with the different viruses at an MOI of 1. At indicated times cellular supernatant from infected NIH3T3 cells (C) and intracellular and extracellular virus from infected BMMΦ (D) were harvested and titrated by standard plaque assays in MEFs. Shown are the median values of 2 independent experiments (n = 3, each experiment) along with SD.

MΦs are a key cellular population for MCMV infection. Furthermore, replication of MCMV in primary bone marrow-derived macrophages (BMMΦ) reflects more closely the *in vivo* phenotype than the replication in fibroblasts [21]. Accordingly, our first experiments sought to characterize the level of infection of MCMVdie1 and parental and revertant MCMV strains in this particular cell population. Therefore, bone marrow (BM) cells were isolated from 10–12 week-old male BALB/c mice for selection of BMMΦ during 7 days of maturation in cell culture before use in infection studies. The F4/80^+^CD11b^+^ phenotype of the BMMΦ was confirmed by flow cytometry analysis prior to infection (Figure S1). First, we determined the virion DNA:PFU ratios for wild-type (referred to as MCMV in this manuscript), *ie1* deficient (MCMVdie1) and revertant-virus (MCMVrev) infection stocks as reported in previous studies [10], [41]. Figure 1A shows that MCMVdie1 develops a similar number of genome equivalents per PFU in comparison with MCMV or MCMVrev. Furthermore, Western blot analysis determining the level of expression of the MCMV early protein E1 24-hrs after infection of BMMΦ further indicated a similar level of early stage-infection of MCMVdie1 and control viruses (Figure 1B). For later stages of infection, the viral growth was determined by standard plaque assays in a single-hit viral growth analysis in the fibroblast cell line NIH-3T3 as well as in BMMΦ (Figure 1C and D, respectively). As expected the results in Figure 1C show that in fibroblasts MCMVdie1 had no defective growth in these cells [11]. However, infection of BMMΦ (Figure 1D) results in a small but significant difference in the growth of MCMVdie1 in comparison with MCMV and MCMVrev. This macrophage phenotype for MCMVdie1 infection is reflective of its *in vivo* phenotype [10], [11] and raises the question of whether this might be due to a possible macrophage-specific pro-inflammatory cytokine response.

### Modulation of BMMΦ cytokine response upon MCMV and MCMVdie1 infection

**Figure 2:**
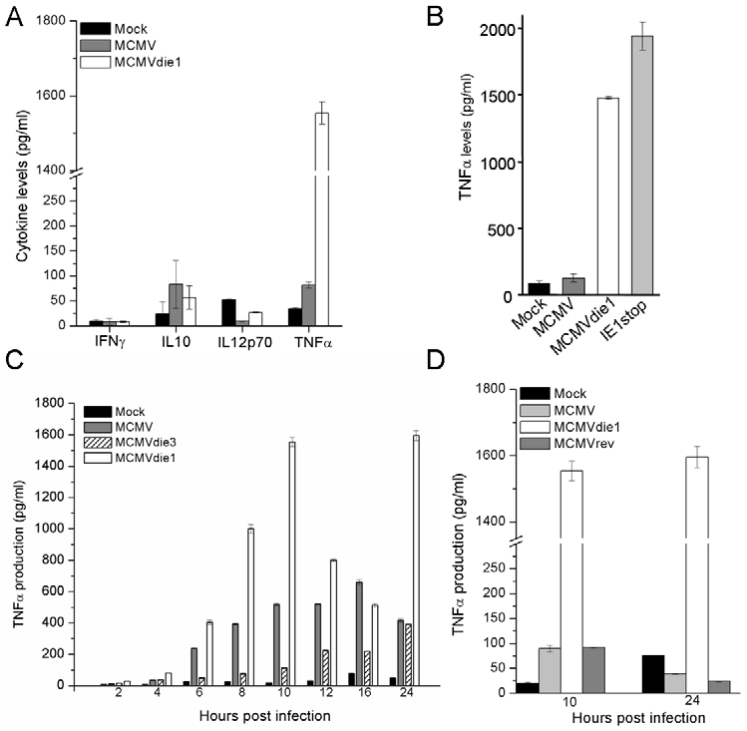
Cytokine production in infected MΦs. (A) Cells were mock-infected or infected with either MCMV or MCMVdie1 (MOI 1). IFNγ, IL10, IL12p70 and TNFα levels from cellular supernatants at 10 hpi were measured by flow cytometry-based CBA. (B) TNFα production after infection of RAW 264.7 macrophages. Cells were either mock-infected or infected with MCMV, MCMVdie1 or the MCMV IE1stop mutant (MOI 1). TNFα levels from the supernatants were determined by ELISA at 10 hpi. (C) TNFα production after infection of BMMΦ with MCMV, MCMVdie1 or MCMVdie3. Cytokine levels from cellular supernatants were measured by flow cytometry based CBA for 12 h time course, 16 and 24 hpi. (D) TNFα production from mock-infected BMMΦ or MCMV-, MCMVdie1- or MCMVrev-infected cells after 10 and 24 h. Cytokine levels were measured by ELISA. Experiments were done in triplicate and tested in duplicates. Bars show mean values with SE.

On the basis of the attenuated growth phenotype of *ie1* null mutant in infection of mice deficient in adaptive immunity (SCID mice), we have previously speculated about a role for IE1 in countering intrinsic or innate cell immunity [11]. As indicated above, infected macrophages characteristically produce a vigorous and varied pro-inflammatory cytokine response, in particular TNFα and are therefore in contrast to other cells, such as NIH 3T3 fibroblast cells that are restricted in their repertoire of cytokine expression. Since *ie1* is known to influence gene expression, it is plausible that IE1 can affect cytokine gene expression in monocyte/MΦ cells. Thus, to directly test the possibility of IE1 protein modulating the cytokine response in infected BMMΦ, we first investigated pro-inflammatory cytokine production after infection of BMMΦ. In these experiments, levels of several cytokines were measured at early times post infection prior to any new infectious virus production. BMMΦ were either mock infected or infected with MCMVdie1 and MCMV at an MOI of 1. Supernatants were harvested at 10 hpi and flow cytometry-based Cytometric Bead Array (CBA) was performed for IFNγ, IL10, IL12p70 and TNFα. As seen from Figure 2A none of the infections differentially altered IFNγ, IL10 or IL12p70 production. However, the TNFα production was only mildly induced by MCMV, but in contrast, MCMVdie1-infected BMMΦs showed a markedly pronounced TNFα response producing a >15-fold higher amount when compared to the levels seen for MCMV-infected cells. It is worth noting that the response can vary due to batch variability in primary macrophages cultures and viral stocks. Nevertheless, similar results have been consistently obtained from multiple independent BMMΦ preparations using different viral stocks.

Furthermore, after infection of a MΦ cell line (RAW 264.7 cells) with MCMVdie1 or another *ie1*-mutant (IE1stop), an approximately 15- to 20-fold higher production of TNFα is also observed in comparison with MCMV (Figure 2B). In the case of the IE1stop mutant the IE1 open reading frame is selectively interrupted, without genetically resecting any further sequences, strongly suggesting that the IE1 protein is responsible for the suppression of the TNFα production and not any other viral protein, that might potentially originate from a the MCMV major IE region.

To further characterise the modulation of TNFα by IE1 during infection we investigated the kinetics of TNFα production. In the following experiments BMMΦ were mock-infected or infected with either MCMVdie1, MCMV or an *ie3* defective MCMV (MCMVdie3) [42] during a 24 h time course. MCMVdie3 is completely defective for viral replication, with viral gene and protein expression essentially restricted to the IE1 and IE2 proteins [41] and thus serves as an excellent comparator for the loss of *ie1* in the MCMVdie1 mutant. Figure 2C shows that after MCMV infection BMMΦ produced detectable levels of TNFα from 4 h onward, until 24 h when it started to decrease. In agreement with the preceding experiment, MCMVdie1 generally induced higher levels of TNFα. Again, the levels of induction found in cellular supernatants from MCMVdie1-infected BMMΦ were higher than those induced by MCMV. It is noteworthy that MCMV does not completely inhibit TNFα production and this may reflect a level of leakiness derived from a pool of low IE1 expressivity of infected cells. Alternatively it could be that low levels of TNFα may be advantageous. In this time course experiment MCMVdie3 moderates the TNFα response with delayed kinetics to the MCMV and MCMVdie1 reaching similar levels as the MCMV by 24 h post-infection (Figure 2C). The observation that MCMVdie3 develops lower levels of TNFα than wild-type MCMV may reflect the importance of an active viral gene transcription that produces double-stranded viral transcripts to induce a full TNFα response. This possibility is consistent with studies showing the role of the cytoplasmic detector for viral RNA RIG-I in inducing and sustaining a TNFα response to infection with a DNA-virus [43]. In such a scenario we would anticipate to see MCMV infection developing higher levels of TNFα than the MCMVdie3. Thus, while an on-going infection is required to trigger a full TNFα response these results appear to indicate that mutation of the *ie1* gene is strongly associated with increased production of the cytokine.

To further evaluate whether the altered production of TNFα after infection with MCMVdie1 is due to the loss of *ie1* gene function and not an accidental second site mutation in the recombinant virus, a revertant virus of MCMVdie1 was also tested. In these experiments BMMΦ were either mock-infected or infected with MCMV, MCMVdie1 and MCMVrev. Supernatants were used to test the levels of TNFα after 10 and 24 hpi. As shown in Figure 2D significant production of the cytokine was found only after infection with MCMVdie1 at both time points, whereas comparably low levels of TNFα were found after infection with MCMV and MCMVrev.

Overall, the viral deletion mutant experiments indicate that the MCMV *ie1* gene plays a previously undisclosed role in moderating the production of a key pro-inflammatory cytokine, namely TNFα, in infected BMMΦ.

### TNFα production is altered in infected cells and not bystander cells

**Figure 3:**
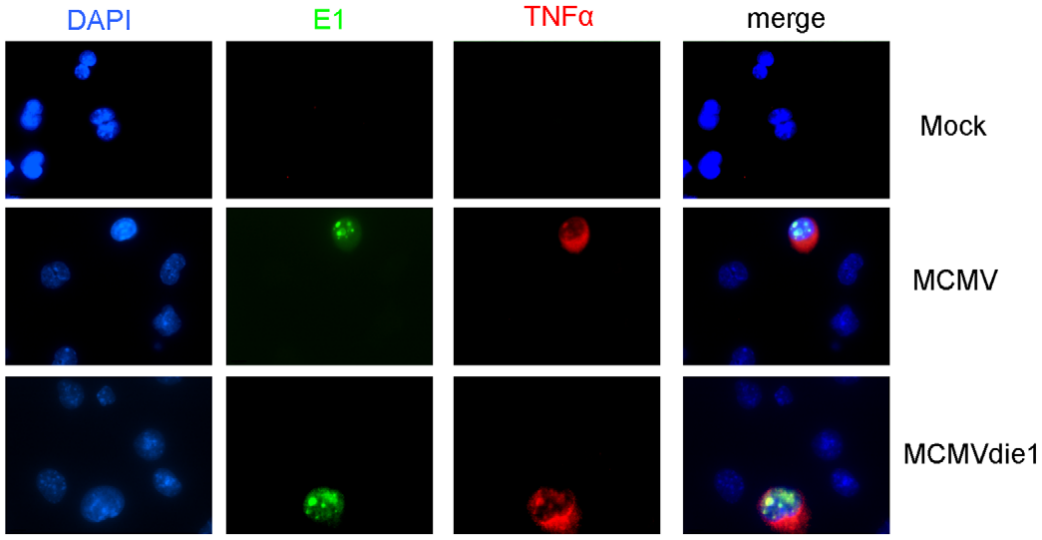
In situ staining of TNFα in viral-infected macrophages. RAW264.7 macrophages were infected with MCMV or MCMVdie1 at an MOI of 1 for 24 h. Cells were fixed with 4% paraformaldehyde and double staining was performed for TNFα and MCMV E1 protein. DNA was counterstained with DAPI.

In our next experiments we sought to determine whether the production of TNFα is specifically associated with the productively infected cells, or bystander cells or both. For these studies immunofluorescence staining monitored the intracellular production of TNFα while staining for viral early E1 antigen simultaneously monitored infection. RAW 264.7 cells were mock-infected or infected with MCMV or MCMVdie1. After 24 hpi, double labelling was performed for MCMV E1 protein and TNFα. As shown in Figure 3, production of TNFα was only detectable in cells that also expressed the E1 protein. LPS was used as a positive control to trigger TNFα production independent of viral infection showing that all cells were competent for TNFα production (Figure S2). These experiments directly show that induced TNFα production is almost exclusively associated with infected cells and not by neighbouring bystander cells.

**Figure 4:**
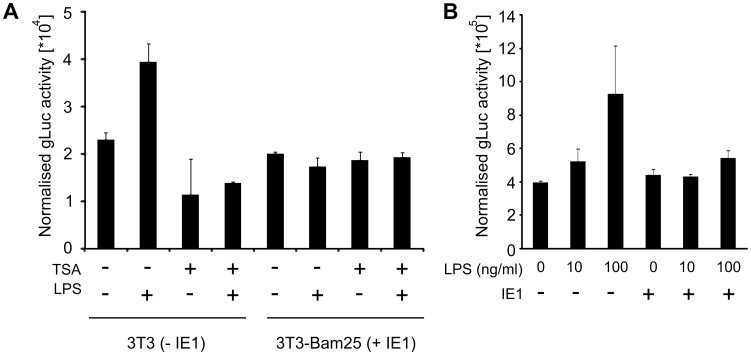
IE1 expression can moderate induced TNFα promoter activity. (A) NIH3T3 cells (−IE1) or Bam25 cells (+IE1) were transfected with 50 ng pTNF-gLuc expression plasmid or pcR3.1 as a negative control. For transfection control 50 ng of pGL3 were co-transfected in all cultures. 24 h post transfection cells were treated for 2 h with 300 nM TSA in PBS or a comparable dilution of DMSO (vehicle) and then LPS (100 ng/ml) stimulated. GLuc activity was measured and normalised to firefly activity per well. Bars show averages (n = 2) of a representative experiment (Error bars = SE). (B) RAW cells were transfected with 125 ng of IE1 expression plasmid pp89UC and 50 ng pGL3 for normalisation. 48 h after transfection cells were stimulated with LPS and normalised gLuc activity was determined. Bars show averages (n = 6) with SE.

These observations raise the possibility that IE1 might play a direct role in moderating the TNFα response in infected macrophages. To explore this possibility and to test whether IE1 alone is capable of moderating TNFα gene expression independent of the infection process, we next used transient transfection assays. To quantify the TNFα promoter activity we constructed a reporter plasmid, pTNF-gLuc, containing the *gaussia* luciferase reporter gene [44], [45] under the control of the murine TNFα promoter/enhancer (position −670 to +1) element. We first assessed the activity of the pTNF-gLuc using the cell line Bam25 [42] that stably expresses the viral IE-genes. For these experiments Bam25 or NIH3T3 cells were transfected with pTNF-gLuc together with the vector pGL3 (Promega) for normalisation of the reporter gene expression measurements. As the infection process results in activation of Toll-like-receptor (TLR) signalling, triggering the expression of pro-inflammatory cytokines, including TNFα [46], we sought to ensure TLR activation in the absence of infection by treating transfected cells with bacterial lipopolysaccharide (LPS). LPS is a known potent inducer for TNFα gene expression [47]–[50]. The transfected cells were incubated for 24 h, culture medium was changed and subsequently stimulated with 100 ng/ml LPS for 4 h. As a control for inhibition of the LPS induced TNFα expression, control cultures were pre-treated with 300 nM Trichostatin A (TSA), an established inhibitor of the TNFα response, for 2 h prior to the LPS stimulation. As shown in Figure 4A, LPS can further induce the normalised reporter gene expression in transfected NIH3T3 cells, while the pre-treatment with TSA inhibits this induction. In contrast to this, LPS stimulation does not induce higher expression levels of *gaussia* luciferase in the Bam25 cell line. These experiments indicate that the expression of the viral IE-genes results in reduced capacity of LPS-induced TNFα activation.

Since the Bam25 cells also express IE3 and IE2 proteins in addition to IE1, we next sought to test if IE1 expression alone is sufficient to restrict LPS-induced reporter gene expression in MΦs. However, transfection of primary MΦs has a low efficiency and therefore we used RAW G9 cells for these experiments. RAW G9 cells were co-transfected with 125 ng of an IE1-expression plasmid, or as a negative control the pcR3.1 cloning vector (Invitrogen), and pGL3 for normalisation. After 48 h incubation medium was changed and cells stimulated with 10 ng/ml and 100 ng/ml LPS respectively. The results shown in Figure 4B reveal that transfection with 125 ng IE1 plasmid was sufficient to block stimulation with both, low and high doses of LPS. These experiments support the suggestion that IE1 alone is sufficient to block LPS induced reporter gene expression.

### IE1 deficient MCMV infection exhibits increased levels of *tnf* gene expression

**Figure 5:**
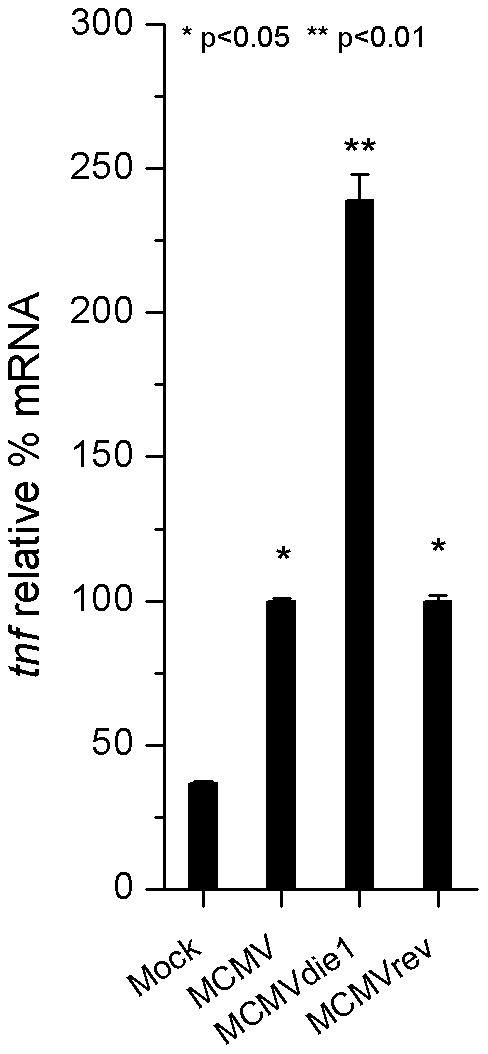
*tnf* gene expression in infected-BMMΦ. Total RNA was extracted at 10 h after mock infection or MCMV, MCMVdie1 and MCMVrev infection of BMMΦ. Quantitative RT-PCR was performed for relative expression levels of *tnf* mRNA in these cells. Shown are relative levels of *tnf* gene expression, which have been normalized against the *gapdh* gene expression and calibrated against MCMV induced-gene expression. SE bars are also shown (n = 3). Significance of differences in mRNA levels in MCMV and MCMVrev infected cells compared to mock infected cells was p<0.05 (*) and for comparison between MCMVdie1 and MCMV or mock p<0.01 (**), respectively (Student's *t*-test).

We next asked whether the absence of IE1 in the context of an infection of BMMΦ also has an effect on TNFα expression at the RNA level. Cells were mock infected or infected at an MOI of 1 with MCMVdie1, MCMV or MCMVrev and harvested for total RNA extraction after 10 hpi. Total RNA was used in quantitative (q)RT-PCR to measure relative *tnf* transcript levels. Data was normalized against *gapdh* levels and the levels of *tnf* from MCMV-infected cells were used as a calibrator. Figure 5 shows relative levels of *tnf* transcripts. When compared to the mock-infected samples, MCMV infection induced expression of *tnf* RNA (p<0.05). However, TNFα mRNA levels in the absence of IE1 protein in MCMVdie1-infected BMMΦ were 2.5-fold higher than those seen for the parental and revertant MCMVs (p<0.01). MCMVrev induction of *tnf* expression was similar to that induced by MCMV. We conclude from these experiments that the viral-induced TNFα production is reduced by MCMV in an IE1-dependent manner in the context of infected MΦs.

### Investigation of IκBα, JNK and p38 kinase signalling in the course of infection

**Figure 6:**
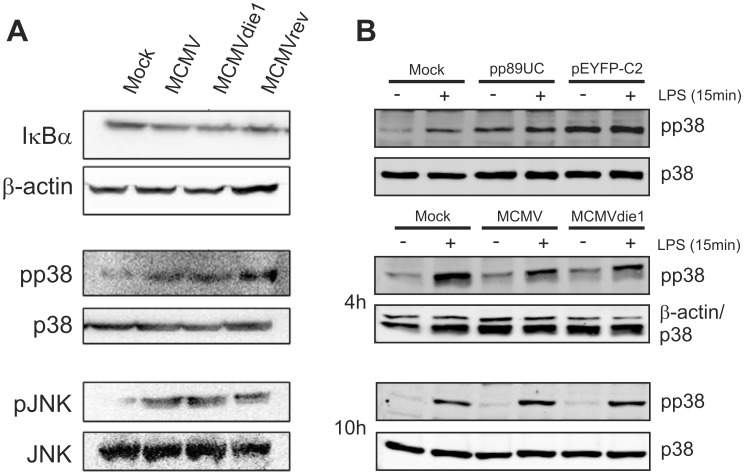
Investigation of IE1-mediated NF-κB, p38 or JNK activation. BMMΦs were mock infected or infected at an MOI of 1 with MCMV, MCMVdie1 or MCMVrev (n = 3). (A) Western blots for IκBα and the activated forms of the p38 and JNK kinases are shown. (B) Western Blots with transfected and infected RAW cells after LPS stimulation. RAW cells were transfected with IE1 expression plasmid pp89UC or a control vector (pEYFP-C2) and stimulated with 10 ng/ml LPS for 15 min or infected (MOI 1) and stimulated at 4 and 10 hpi, respectively.

TNFα transcription is induced by different stimuli in a cell-type dependent manner [28], [51]–[54]. The key signalling molecules, NFκB, JNK and p38 MAPK are a common set of targets by which microbial and fungal pathogens inhibit TNFα production [28], [37], [38], [40]. However, in quantitative Western blot experiments involving infection of BMMΦs with MCMV, MCMVdie1 or MCMVrev analysis of IκBα, p38 and JNK proteins and their phosphorylated forms (Figure 6A) reveals that at 10 hpi there are no significant differences between the three different viruses tested (for quantification see Figure S7). All three viruses induce activation of both p38 and JNK kinases but not IκBα. The absence of IE1 upon infection did not result in a differential change in the level of IκBα or in the expression or phosphorylation levels of the tested kinases at 10 h post infection. It is possible that the time point analysed is too late to detect any quantitative differences and that they may be temporally masked by a range of cross-talking signalling events during the infection process.

While lack of activation of NFκB by MCMV is consistent with our previous studies it is possible that its activation is only detectable at IE times of infection. For the purpose of exploring whether differences in the activation of NFκB may be observable at more immediate early times we have used a stably transfected cell line, RAW G9 cells [55], to visualise the activation of NFκB. These cells express the NFκB subunit p65 as a GFP fusion protein. In rested cells the NFκB complex is distributed throughout the cytoplasm, producing a weak and diffuse GFP signal in the cells (Figure S4, mock treated sample). After LPS stimulation NFκB is activated and translocates to the nucleus, leading to an enrichment of GFP in the nucleus. The translocation of p65-GFP to the nucleus from the cytoplasm consequently develops a visibly concentrated nuclear GFP signal. We therefore infected RAW G9 cells for 45 min and observe enrichment in nuclear fluorescent cells for LPS but not for MCMV and MCMVdie1 infected samples (Figure S4). Quantification of the level of cytoplasmic and nuclear fluorescence indicates similar levels for MCMV and MCMVdie1. This indicates that MCMV and MCMVdie1 are equally restrictive for NFκB signalling during the IE1-phase of infection.

As noted in the previous section the expression of IE1 in the transient transfection reporter assays appears to have a similar inhibitory effect on the LPS-induced activity of the TNFα- promoter as the HDAC inhibitor TSA (Figure 4A). The mechanism for the TSA-mediated inhibition of TNFα activation is known to work through inhibiting p38 phosphorylation in RAW 264.7 cells [56]. Therefore we sought to test if IE1 in the transient transfection assay can interfere with the phosphorylation of p38 after LPS stimulation. RAW cells were either left untreated or were transfected with pp89UC or pEYFP-C2, an YFP expressing control vector. 48 h after transfection cells were stimulated with LPS for 15 min and pp38 levels were analysed by quantitative western blot analysis (Figure S7). Figure 6B shows, as expected, that treatment of mock-transfected cells with LPS leads to an increase in p38 phosphorylation. Notably, transfection with the IE1 plasmid (pp89UC) led to a high level of p38 phosphorylation that could not be further increased by subsequent LPS stimulation. In our system, the inability of LPS to induce higher levels of pp38 in IE1-expressing cells is not due to an exhaustion of the p38 pool, since transfection with the YFP control plasmid led to ∼1.4-fold higher signal for pp38 than the transfection with pp89UC (Figure S7). These experiments therefore indicate that IE1 might impart the ability to restrict the phosphorylation of p38.

We next sought to extend this observation to the infection system at IE-times of infection. We compared the induction of p38 phosphorylation by LPS (15 min) in infected RAW cells at 4 and 10 hpi in comparison to mock-infected samples. As shown in Figure 6B the treatment of control cultures with LPS induces the phosphorylation of p38. In contrast to this the virus infected samples showed a slightly weaker signal for pp38 than the mock infected samples at 4 hpi, indicating that the virus might be capable to moderate the phosphorylation of p38. MCMVdie1, however, showed no increased phosphorylation over the MCMV infection for both the non-stimulated and the LPS stimulated samples. Normalisation of the pp38 signal to the levels of p38 protein revealed that there was no detectable difference in levels of pp38 in the LPS stimulated samples (Figure S7). In accordance to this we found no increase in levels of phosphorylated p38 at 10 hpi. Under these experimental conditions quantification of the western blot and normalisation to p38 abundance actually revealed that treatment of MCMVdie1 infected cells induced slightly less pp38 compared to MCMV infected samples (Figure S7). To also check for differences in phosphorylation of JNK we subsequently determined the levels of pJNK on the same membranes and found that MCMV and MCMVdie1 induced the same level of pJNK signal as stimulation with LPS (Figure S7).

Altogether these experiments provide evidence against a mechanism involving IE1 inhibition of p38, NFκB or JNK signalling for the induction of TNFα in the context of an infected macrophage.

### Role of IE1 in moderating TNFα transcript levels *in vivo* in the immune compromised host model of CMV disease

**Figure 7:**
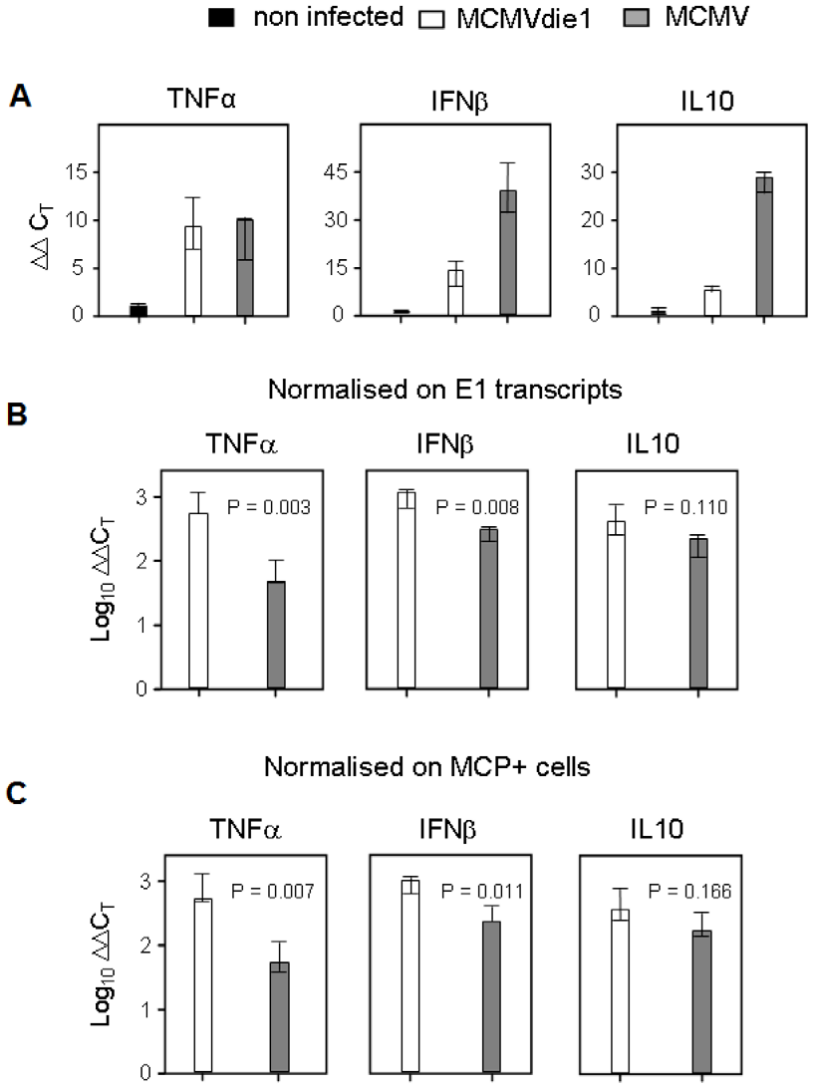
In situ activation of cellular genes TNFα, IFNβ and IL10 during infection of the liver. Immune compromised female BALB/c mice (n = 3 per group) were infected with 1×10^5^ PFU of MCMV or MCMVdie1 or not infected, i.e. uninfected but immune compromised mice to take into account that a 6.5 Gy total-body γ-irradiation by itself slightly stimulates the expression of the genes under investigation. The analysis was performed 10 days after infection. (A) Expression levels (ΔΔC_T_ values) relative to β-actin transcripts. (B, C) Normalisation of the relative expression levels of TNFα, IFNβ, and IL10 to the numbers of E1 transcripts per 500 ng of total RNA (B) or to the numbers of infected MCP^+^ cells per representative 10-mm^2^ areas of liver tissue sections (C) in order to take account of differences in tissue infection density. Throughout, bars represent median values for three mice per experimental group. Variance bars indicate the range. P values for significance are indicated for group comparisons of interest (unpaired *t*-test, two-sided, performed with log-transformed data).

While modulation of TNFα by a wide range of pathogens has been extensively studied in cell-culture (reviewed in [28]), it remains to be determined whether such modulation occurs *in vivo*. Accordingly, biological significance of cell culture-based observations should be treated with some caution. Thus, to directly determine the physiological significance played by IE1 in moderating TNFα, we next studied the role of MCMV IE1 protein *in vivo*. As suggested above for BMMΦs in cell culture, IE1 modulates TNFα expression already at the transcript level. We therefore tested if this applies also to TNFα transcription in host organs relevant to CMV pathogenesis. The experiment was performed in immune compromised, γ-irradiated BALB/c mice, an established model for lethal, multiple-organ CMV disease [57]. In this model it was demonstrated previously [10] that wild type MCMV replicates in the liver with a doubling time of ∼19 h, whereas MCMVdie1 was found to be growth-attenuated with a doubling time extended to ∼34 h. Likewise, MCMVdie1 was found to be growth-attenuated also in spleen and lungs [10]. As suggested above for BMMΦs in cell culture, IE1 modulates TNFα expression. We therefore tested if this applies also to TNFα in the liver by quantitating steady-state levels of *tnf* transcripts with qRT-PCR. As seen in Figure 7A, TNFα gene expression in both infected groups is detectable above the baseline defined by uninfected liver tissue. At first glance, one might conclude that the TNFα gene expression level is modestly higher in MCMV-infected livers compared to MCMVdie1. For correctly interpreting the gene expression data, however, one must consider the different viral burden in tissue due to the growth-attenuation of the *ie1*-deletion mutant [10]. To account for this problem, we normalized TNFα gene expression to the number of viral E1 transcripts determined by qRT-PCR (Figure 7B). As shown in Figure 1 expression levels of E1 were comparable in the infected BMMΦs in the *in vitro* system. However, in the more complex *in vivo* system it is unclear at which level this impairment is manifest in the viral replication cycle. To exclude that an impaired E- or L-gene expression could influence our normalisation strategy for the qRT-PCR, we sought to use a method for normalisation that is independent of transcript levels in infected cells. To normalise for the different levels of infection in the sampled organs we therefore directly measured the number of infected cells by *in situ* immunostaining for the late major capsid protein (MCP, M86) (Figure 7C). Regardless of which normalization strategy was used, the normalised expression level of TNFα was found to be ∼10-fold higher in MCMVdie1-infected livers than in MCMV infected organs. For comparison, normalized gene expression levels of the cytokines IFNβ and IL10 were measured in order to further probe the role of IE1 in regulating the innate cytokine response. Interestingly, infection with the *ie1*-deletion mutant also induced significantly higher amounts of IFNβ transcripts (Figure 7B and C) as compared to MCMV. In accordance with the IL-10 expression in BMMΦs (Figure 2A), IL10 transcription in the liver was induced by the infection (Figure 7A) but was not significantly influenced by IE1 (Figure 7B and C). Altogether, TNFα, and to some degree also IFNβ, are suppressed on the transcript level in an *ie1*-dependent manner *in vivo* at a relevant organ site of CMV disease.

### MCMV IE1 modulation of TNFα protein levels *in vivo*

**Figure 8:**
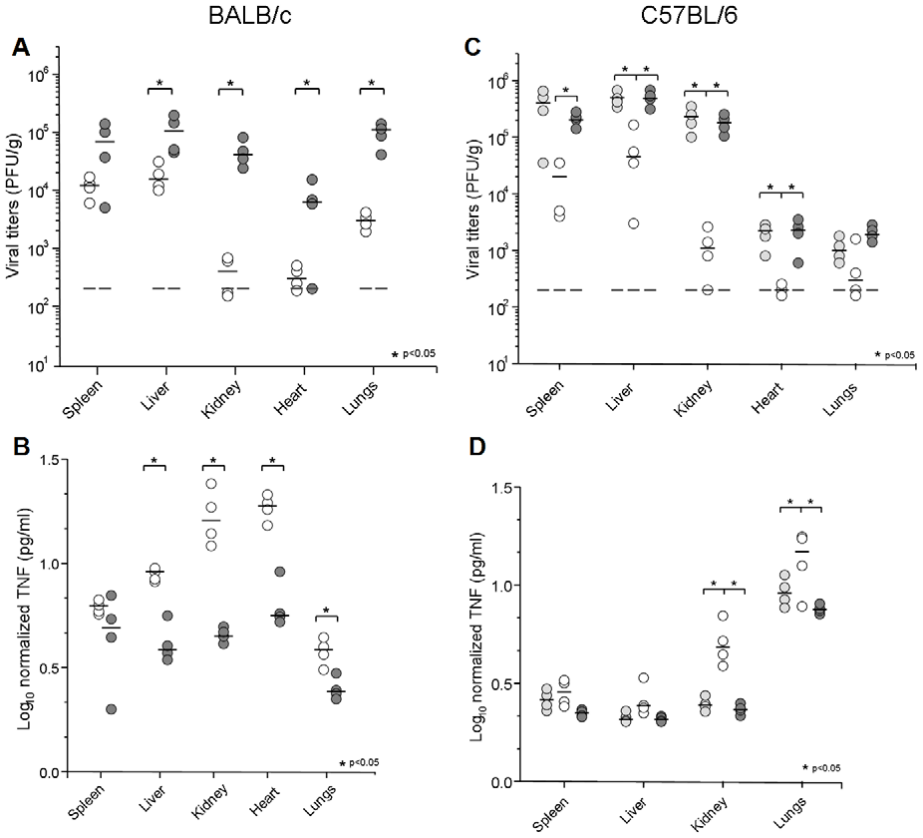
Growth of MCMV, MCMVdie1 and MCMVrev and TNFα production in different organs from infected mice. BALB/c (A, B) or C57BL/6 mice (C, D) were infected with 3×10^6^ PFU or 2×10^6^ PFU, respectively, by the i.p. route. Groups of mice (4 to 5 mice per group) were inoculated with the specified dose of parental MCMV (light grey circles), MCMVdie1 (open circles) or MCMVrev (dark grey circles). On day 4 post infection mice were killed and the indicated organs were harvested, weighted, and sonicated as a 10% (wt/vol) tissue homogenate in DMEM. (A and C). Viral titres were determined by standard plaque assay on MEFs. Dashed lines show limit of detection. Dark horizontal marks show median values. (B and D). Ratios between TNFα levels and PFU in different organs at day 4 after infection are shown in a log scale. Statistically significant differences in viral titres and TNFα levels between MCMVrev or MCMV and MCMVdie1 are indicated by an asterisk (p<0.05).

We next determined if transcriptional control by IE1 also translates into TNFα protein levels in host tissues. In a first set of *in vivo* experiments, groups of 4 to 5 BALB/c and C57BL/6 immune competent mice were infected by the i.p. route, with the viral mutant and control strains. Viral yields and TNFα levels were determined at day 4 p.i. for selected organs. As seen in Figure 8 A and C, MCMVdie1 showed the expected attenuated phenotype in both mouse strains when compared to MCMV and MCMVrev [11]. Viral titres in spleen, liver, heart and lung from infected BALB/c mice were 6-, 12-, 21- and 35-fold reduced, respectively. The attenuation was even more dramatic in kidneys where the MCMVdie1 titres were reduced by a factor of >100. In C57BL/6, as in BALB/c mice, both MCMV and MCMVrev exhibited comparable replication (Figure 8C). MCMVdie1 titres were significantly reduced in all organs examined with a 10-fold reduction in spleen and liver and over 100-fold reduction in kidneys. From this data we conclude that the attenuation shown by MCMVdie1 is not mouse strain-dependent, since in BALB/c, C57BL/6 and also in the 129 strain (data not shown) as well as in γ-irradiated mice [10], the *ie1*-deletion mutant MCMVdie1 is not able to replicate as efficiently as MCMV or MCMVrev.

As described above for the RNA expression studies, MCMV induces TNFα production in an acute infection [58]–[60], and it is understood that TNFα levels positively correlate with the level of infection [59]–[61]. In agreement with these previous studies we also observed a positive correlation between TNFα protein levels and infectivity per gram of tissue in kidneys and heart (Figure S3). On this basis, TNFα was quantified and cytokine levels were calibrated against viral titres in those samples where infectious virus was detectable. Accordingly, normalized TNFα levels are shown in Figure 8B and D. At day 4 p.i., MCMVdie1 induces by an order of magnitude higher levels of TNFα than those detected for MCMVrev in all organs tested except in the spleen of both BALB/c (C), and C57BL/6 mice, and liver of C57BL/6 mice (D).

Taken together, these results support a role of IE1 in moderating the production of TNFα *in vivo*.

**Figure 9:**
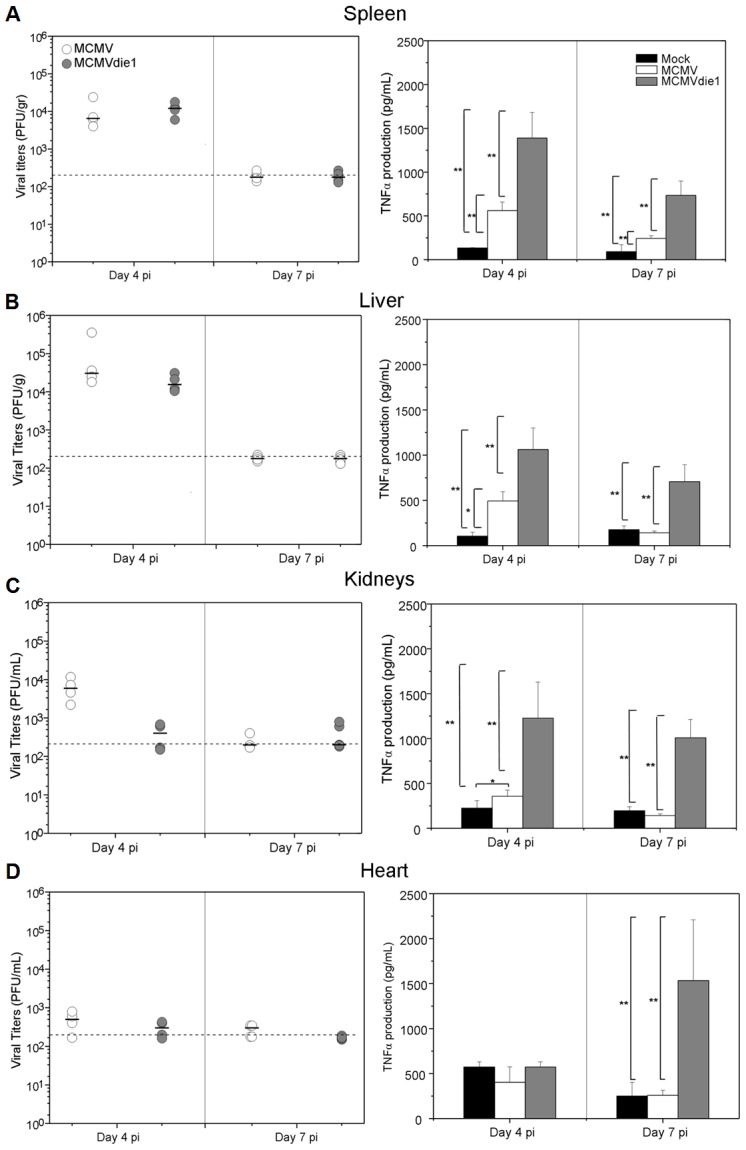
Growth of MCMV and MCMVdie1 and TNFα levels in different organs of infected BALB/c mice. Groups of BALB/c mice (4 mice per group) were inoculated with either 3×10^5^ or 3×10^6^ PFU of parental MCMV or MCMVdie1, respectively. On day 4 and 7 post infection mice were killed and spleens, livers, kidneys and hearts (A, B, C and D, respectively) were harvested, weighted, and sonicated as a 10% (wt/vol) tissue homogenates in DMEM. **Left panels.** Viral titres were determined by standard plaque assay on MEFs. Grey lines show limit of detection. Black horizontal marks show median values. **Right panels.** Levels of TNFα in different organs of infected BALB/c mice. At indicated times organs were harvested, weighted and homogenated as a 10% (wt/v). TNFα levels were determined by ELISA from the homogenates. Mock infected mice were also included as a negative control. No significant differences were found in production of infectious virus between infections, except in kidneys at 4 dpi. Statistically significant differences in TNFα levels are shown as * = p<0.05 or ** = p<0.01.

Because of the attenuated phenotype of the *ie1*-deletion mutant, the virus titres per gram of tissue differ significantly between infected groups. It is therefore possible that an IE1 expressing virus is simply able to replicate more efficiently and consequently will block TNFα production by alternative mechanisms. We therefore sought to establish an equivalent level of infection (PFU per gram of tissue) by adjusting the initial infectious dose of the inoculums and determining TNFα production. In the following experiments, immune competent BALB/c mice were infected with 3×10^5^ PFU of MCMV or 3×10^6^ PFU of MCMVdie1, and both viral titres and the absolute TNFα response were compared in different organs at days 4 and 7 post-infection. Under such conditions, viral titres of MCMV and of the *ie1*-deletion mutant were found to be comparable in all organs (Figure 9, left panels), except in the kidneys (Figure 9C) at day 4 p.i. In comparison to day 4 p.i. the observed reduction in titres by day 7 p.i, is indicative of the clearance of the virus from these various organs. TNFα levels after infection were measured in the different organs from viral-infected and mock-infected mice (right panels). Results show that MCMV induced TNFα production after 4 days of infection in all organs tested, except in the heart (Figure 9D). Importantly, there was also a marked increase in the levels of TNFα between MCMV and MCMVdie1. In general, the mutant virus induced statistically higher levels of TNFα in spleen, liver and kidney after 4 days of infection; however, there was no difference in the heart (Figure 9D). At one week of infection, viral clearance of both MCMV and MCMVdie1 has mostly taken place in all organs tested with the levels of TNFα having returned to mock levels except for a residual higher level in the spleen for the MCMV infection (Figure 9A). Strikingly, and in stark contrast to the MCMV infection, TNFα levels remained elevated in MCMVdie1 infected animals.

Altogether these results show that the production of TNFα can be significantly moderated by an *ie1*-dependent mechanism in an acute *in vivo* infection by MCMV.

### TNFα is sufficient to inhibit MCMVdie1 *in vitro* but is not essential for inhibition *in vivo*

**Figure 10:**
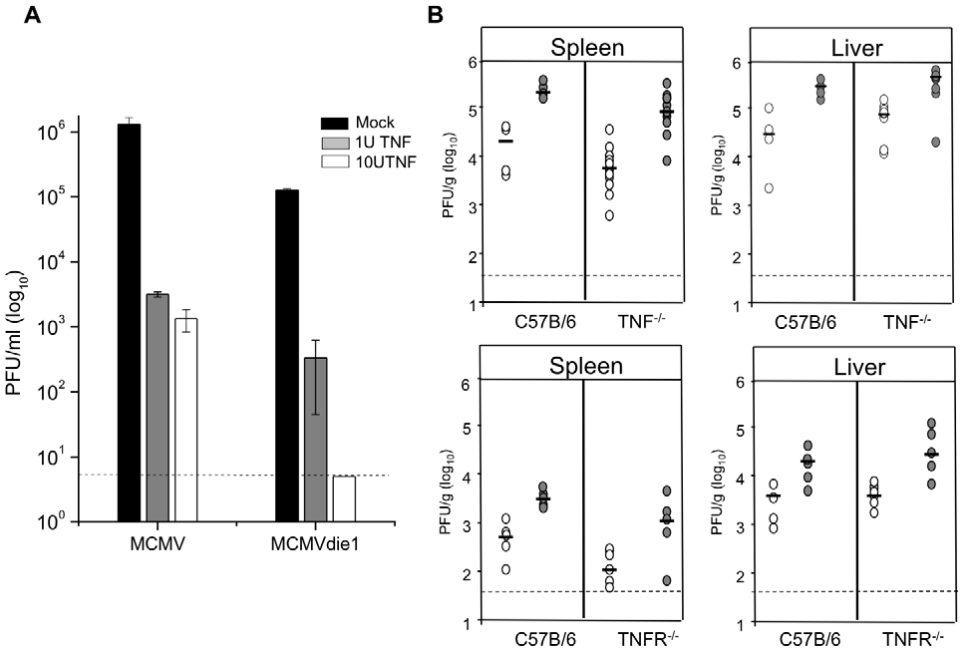
TNFα blocks viral replication *in vitro* but is not essential *in vivo*. (A) BMMΦs were pre-treated for 24 h with TNFα as indicated and subsequently infected with either MCMV or MCMVdie1 (MOI 1). Total virus was measured by plaque assay at day 3 p.i. and dashed line indicates limit of detection with bars showing averages (n = 3) with SE. (B) Organs from infected C57B/6, TNFα^−/−^ or TNFRp55^−/−^ mice (2×10^6^ PFU i.p.) were harvested at 4 d p.i. and homogenated for analysis with standard plaque assay (MCMV = grey circles; MCMVdie1 = open circles). Titres were normalised per sample weight, black lines indicate median values and dashed line represents limit of detection.

It has been shown by others that despite the protective effects of TNFα pre-treatment *in vitro* an *in vivo* pre-treatment does not increase survival rates of MCMV infected mice [62]. We therefore first sought to determine the effect of TNFα on MCMV and MCVMdie1 replication in macrophages *in vitro*. Differentiated macrophage cultures were pre-treated with 1 U/ml or 10 U/ml recombinant mouse TNFα (Endogen, PIERCE; 10 µg/ml corresponded to 10^5^ U/ml) for 24 h prior to infection and viral replication monitored by plaque assay at day 3 p.i. As expected Figure 10A shows that pre-treatment with TNFα *in vitro* reduces viral titres of MCMV. Notably TNFα has a much stronger effect on the titres of MCMVdie1, which are reduced to the limit of detection, indicating that MCMVdie1 is more susceptible to TNFα control compared to MCMV *in vitro*.

We next sought to determine the contribution of TNFα in controlling viral replication *in vivo* by using mouse strains deficient either in TNFα expression (TNF^−/−^) or TNFα signalling (TNFRp55^−/−^). For these investigations groups of mice were infected with either MCMVdie1 or MCMVrev as indicated in Figure 10B and viral titres were measured in organs at day 4 p.i. to investigate if abrogation of TNFα expression or signalling could enhance or rescue the replication of MCMVdie1. In both tested mutant mouse strains the impairment of TNFα expression (TNF^−/−^) or TNF signalling (TNFR^−/−^) did not rescue impaired replication of the MCMVdie1 in the livers, spleens or other organs analysed (see Figure S5). To complement this experiment and to control for side effects of the genetic ablation of TNFα we also analysed the effects of antibody-mediated depletion of TNFα in vivo. Mice were infected with equal doses of MCMVdie1 and MCMVrev and anti-TNFα antibodies were injected at day 0 and 2 p.i. As shown in Figure S6 at 4 dpi no significant differences between the MCMV and MCMVdie1 viruses could be detected in all analysed organs.

Together these results show that while TNFα is sufficient to inhibit MCMVdie1 *in vitro* it is not essential for inhibition *in vivo* and may indicate that in the more complex *in vivo* setting other host factors can complement for the loss of TNFα function.

## Discussion

In this study we have presented evidence revealing that a viral gene, *ie1*, of MCMV is involved in altering the pro-inflammatory cytokine response, in particular TNFα production *in vitro* and *in vivo*. Our results not only identify a new and previously undisclosed functional role for IE1 in moderating the inflammatory response to infection, but also show for the first time the association of a specific pathogen-encoded gene to restrict TNFα protein and RNA production *in vivo* in the context of a natural infection.

Several pathogens have been shown to modulate TNFα-induced response by a wide range of different mechanisms. For instance, direct interaction with TNFα has been reported for the M-T2 protein of the Myxoma virus avoiding TNFα-TNFR interaction [63]. African swine fever virus also targets TNFα-induced gene expression in infected-MΦs by a mechanism involving the viral protein A238L [64]. Inhibition of TNF receptor has also been shown for Adenovirus and Poliovirus [65], [66]. Moreover, HCMV and MCMV have also been shown to down modulate TNF receptor expression [29]–[31], blocking TNFα-induced gene expression in infected cells. However, there are only a few examples in the literature of viral proteins that interfere with TNFα production [36]. From the literature it can be seen that targeting the production of TNFα is a common feature in microbial infections, as seen for *Salmonella*, *Yersinia* and *E. coli* [37]–[40]. All known mechanisms by which microbial pathogens alter the TNFα production involve targeting p38 MAPK and JNK kinases and/or NF-κB activation (reviewed in [28]). Here, we demonstrate that the viral IE1 protein of MCMV moderates TNFα production in infected BMMΦs. Moreover, although the exact mechanism by which IE1 exerts its effect on TNFα production remains open, our experiments studying the contribution of key signalling molecules p38 MAPK, and JNK, as well as NF-κB, suggest that IE1-induced modulation of TNFα production does not involve altering the function of these signalling proteins. Although, we do not exclude that the phosphorylation of JNK or other phosphatases is also influenced by other viral factors and therefore effects of IE1 deletion on phosphorylation of these signalling factors could be masked.

Comparison of TNFα levels between MCMV and the MCMVdie3 mutant virus showed that MCMVdie3 infection produces much lower levels of TNFα compared to MCMV infection until 24 hpi. This indicates the importance of viral gene transcription for inducing and sustaining a full scale TNFα response. This observation is in good accordance with evidence showing that RIG-I mediated detection of Myxoma virus transcripts in human macrophages is necessary to induce a full TNFα response [45]. Furthermore we show that MCMV does not completely inhibit TNFα production. It has been demonstrated that CMV can reduce the effects of TNFα by down-regulation of the TNFα receptor during infection [29] and thereby increasing tolerance to extra-cellular TNFα levels. A major factor determining permissiveness of cells of the myeloid-monocytic lineage for infection with CMV is the state of maturation ([67] and references therein) and it has been demonstrated that TNFα also facilitates maturation of macrophages [68]. In addition TNFα can be pro-viral by initiating infection through signalling to the MIE enhancer. Taken together this indicates that low levels of TNFα may well be tolerated by CMV and could under certain conditions also have a pro-viral effect. Thus it may not be to the best advantage for CMV to completely abrogate TNFα production.

IE1 has been described to have a very general effect on gene transcription levels including more genes than are controlled by p38 signalling directly and is better known for its intra-nuclear activities including the disruption of ND10 bodies [15], [69]. In the case of HCMV, the ability of IE1 to disrupt ND10 bodies has been correlated with the disruption of an intrinsic defence mechanism involving nuclear repressor proteins, such as hDaxx and histone deacetylases (HDACs), to inhibit viral immediate-early gene expression [17]. In case of MCMV, a direct interaction of IE1 with Daxx and HDAC2 has been demonstrated [12] in good accordance with the general induction of transcription caused by IE1 [9], probably involving de-repression of chromatin. It has been demonstrated that expression of TNFα is partially regulated by chromatin re-modelling and that treatment with TLR ligands such as LPS initiates de-repression of chromatin in macrophages [47], [48]. Therefore it is possible that the influence of IE1 on HDAC function could interfere with TNFα expression by disrupting the de-repression or maintaining repressive chromatin associated with the TNFα promoter. It remains to be determined how IE1 precisely inhibits TNFα expression while also potentially inducing a de-repression of chromatin.

It is noteworthy that Trichostatin A (TSA), which we used as a control reagent to block LPS induced TNFα gene expression [56], is also an inhibitor of HDACs and therefore leads to a general de-repression of chromatin, while it is capable of inhibiting TNFα expression [50], [56]. This presents parallels between TSA and IE1 and allows speculating that these molecules work potentially through a similar mechanism. A known mechanism for the effect of TSA on TNFα is mediated through the p38-inhibtor MAPK phosphatase-1 (MKP-1). In this case the stability of the interaction of MKP-1 with its target molecule, p38 MAPK, is regulated by the acetylation of the interaction site, which is negatively regulated by HDACs. Treatment with the HDAC inhibitor TSA increases the level of the acetylated form of MKP-1 and thereby links the inhibitory function of MKP-1 on the p38 signalling pathway to HDAC activity [56]. In accordance with this it has been furthermore demonstrated that LPS stimulation activates HDAC-3 [50] and is also known to induce expression of HDAC members in BMMΦs [70]. This would negatively regulate MKP-1 activity, increasing p38 signalling and therefore TNFα expression. Since it is established that IE1 interacts and inhibits HDAC activity [12] it raises the question if IE1, at least partially, could function through this mechanism.

In this scenario we would anticipate increased pp38 levels in MCMVdie1-infected cells. However, we do not detect any increase in pp38 levels in MCMVdie1 infected cells compared to MCMV in the first 10 hpi and thus strongly indicating that IE1 does not interfere with p38 phosphorylation in the context of infection. Although notably, studies have shown that changes in acetylation of proteins can take up to 9–11 h to occur in the case of histones [71]. This might indicate that IE1-mediated changes on MKP-1 acetylation become apparent at later stages of the infection and could therefore have a role in the late phase of replication, complementing other viral factors interfering with TNFα action, such as M45 [29], [31]. A more thorough investigation of MKP-1 and its effects on MCMV replication and induction of TNFα will be necessary to clarify if IE1 inhibits p38 signalling.

However, it is also noteworthy that the immune-modulatory cytokine IL10 is induced upon infection by MCMV in MΦs and *in vivo* and since IL10 is known to function as an anti-inflammatory mediator, it is possible that a host IL10 autocrine loop might be involved in modulating TNFα. While we cannot exclude the possibility for IL10 involvement in TNFα suppression we failed to detect any significant difference in expression of IL10 between MCMV with and without *ie1* indicating that the mechanism for TNFα moderation does not involve regulation of IL10.

While many pathogens have been shown to counter-regulate TNFα production in cell culture, in particular in MΦs, little or no information is available to indicate whether this mode of regulation also occurs in an *in vivo* system. Our investigations show that the viral gene, *ie1*, in the context of a natural infection contributes to a significant moderation of TNFα production in multiple organs *in vivo*. TNFα levels, relative to the amount of detected virus, in selected organs were significantly higher in animals infected with MCMVdie1 than in animals infected with MCMV or MCMVrev, and this was consistently observed in several genetic backgrounds and in different infection models. This is quite striking, as we would anticipate a significantly reduced inflammatory response to be associated with the severely attenuated *in vivo* growth by MCMVdie1. *In vivo* we observed a relatively enhanced production of the pro-inflammatory cytokines TNFα and IFNß but not for the anti-inflammatory cytokine, IL10, with MCMVdie1 infection. Because of the attenuated phenotype of the *ie1*-deletion mutant, the virus titres per gram of tissue differ significantly between infected groups. It is therefore possible that an IE1 expressing virus simply is able to replicate more efficiently *in vivo* and consequently blocks TNF production through alternative methods. While we have not investigated in the present study other similarly attenuated viruses in their ability to develop an elevated pro-inflammatory cytokine response, we have evaluated TNFα production levels in the context of establishing an equivalent level of infection per organ between the *ie1* deficient and wild-type strains. These studies clearly indicate that even in the presence of comparable levels of infection, MCMVdie1 induces significantly higher levels of TNFα. Those levels were sustained for at least 1 week after infection, whereas wild-type MCMV-induced TNFα production had returned to the mock-infected levels by 7 days p.i. The results of our investigation provide the first demonstration of a counter-regulatory role encoded by the *ie1* gene in moderating the TNFα cytokine production in the context of a natural infection *in vivo*. In accordance with the attenuated *in vivo* phenotype of MCMVdie1 we could demonstrate a higher sensitivity of MCMVdie1 replication *in vitro* compared to MCMV but neither genomic deletion of the TNFα gene, the TNFα receptor gene or in vivo depletion of TNFα by administration of anti-TNFα antibodies could rescue the attenuated MCMVdie1 phenotype, indicating that other anti-viral host factors complement the loss of TNFα function. In accordance to this we find a slight but significant increase in IFNb1 expression in infected organs.

In summary, we identify a new biological role for the *ie1* gene of MCMV involving the regulation of pro-inflammatory cytokines, especially TNFα production, in both *in vitro* and *in vivo* infections. This suggests a novel viral counter-immune strategy preventing a robust inflammatory response during an infection. Our findings demonstrate that IE1, in addition to blocking intrinsic cell defences, also acts as a virulence factor contributing to viral modulation of the inflammatory response. This new role of *ie1* may also have therapeutic anti-viral and anti-inflammatory applications in the future. In this regard, our results provide the first evidence of a viral strategy capable of suppressing pro-inflammatory TNFα production *in vivo* mediated by a pathogen-encoded gene.

## Materials and Methods

### Ethics statement

All procedures involving animals and their care were approved by the Ethics Committee of the University of Barcelona (Spain) and were conducted in compliance with institutional guidelines, as well as with catalan (Generalitat de Catalunya decree 214/1997, DOGC 2450) laws and policies. In the United Kingdom experiments were approved by the University of Edinburgh Animal Procedures and Ethics Committee and were performed under licence from the United Kingdom Home Office under the Animals (Scientific Procedures) Act 1986. In Germany, mice were bred and housed under specified pathogen-free conditions in the Central Laboratory Animal Facility of the Johannes Gutenberg-University, Mainz. Animal experiments were approved according to German federal law under permission number 177-07-04/051-62. In Croatia all animals were bred and housed at the Breeding Facility of the Faculty of Medicine, University of Rijeka. Experiments were approved by the Ethics Committee of the University of Rijeka and were performed in accordance with the Croatian Law for Protection of Laboratory Animals (matched with EU legislation (DIRECTIVE 2010/63/EU OF THE EUROPEAN PARLIAMENT AND OF THE COUNCIL of 22 September 2010 on the protection of animals used for scientific purposes).

### Cells and viruses

The murine fibroblast cell line NIH3T3 cells (ATCC CRL1658) and the macrophage cell line RAW 264.7 (ATCC TIB-71) were obtained from the American Type Culture Collection (Manassas, VA). Primary murine embryonic fibroblasts (MEFs) were prepared from embryos of pregnant BALB/c mice on day 16 of gestation. RAW G9 cell line was constructed as described previously [55] and expresses a p65-GFP fusion protein under transcriptional control of the native p65-promoter. NIH3T3, were cultured in Dulbecco's modified Eagle's medium (DMEM) supplemented with 10% calf serum (CS) and RAW 264.7, RAW G9 and MEFs were cultured in Dulbecco's modified Eagle's medium (DMEM) supplemented 10% fetal CS (FCS). All cell media also contained 2 mM glutamine and 100 U of penicillin/streptomycin per ml. The *ie1*-deficient mutant (MCMVdie1) and corresponding revertant virus were described in [11]. The IE1stop mutant carries a stop codon at the 5′-end of exon 4 of the MCMV *ie1* gene preventing the synthesis of the pp89 IE1 protein. Briefly, two successive PCR rounds were performed using the primer pairs IE1_stop_fw1 (5′-GCAATCTTACAGGACAACAGAACGCTCTACACTGGAGGATGACGACGATAAGTAGGG-3′) and IE1_stop_rv1 (5′-TACAAACCACTCTTATATTCCAGTGTAGAGCGTTCTGTTGCAACCAATTAACCAATTCTGATTAG-3′), and IE1_stop_fw2 (5′-GATTGATAGTTCTGTTTTATCATGAGGTGTGCAATCTTACAGGACAACAGAACGCTCTACACTGGA-3′) and IE1_stop_rv2 (5′-CTGTCTTTCATATTCACCCACACAGAACACTTGAGTTATACAAACCACTCTTATATTCCAGTGTAGAGCG-3′), respectively, to amplify a kanamycin resistance marker, followed by recombination in *E. coli* using the MCMV BAC pSM3fr [72] and applying the *en passant* mutagenesis procedure as described in [73] The parental BAC-derived MCMV strain MW97.01 ([72], named MCMV in this study) and the recombinant MCMVs were propagated on NIH3T3 cells. Cells were grown to 70%–80% confluency and infected at an MOI of 0.01 with MCMV, MCMVdie1 and MCMVrev in DMEM supplemented with 2% CS. When cultures reached cytopathic effect, supernatants were harvested and kept at −70°C after clearing cellular debris. Viral titres were determined by standard plaque assays on MEFs. Bone marrow derived macrophages (BMMΦ) were prepared from 10–12 week-old male BALB/c mice as described previously [74]. Femur lavages were plated out at 5×10^5^ or 8×10^5^ cells per well (24- and 6-well plates, respectively) and left for maturation for 7 days in DMEM:F-12 containing 10% FCS, 10% L929 conditioned media as a source of macrophage colony stimulating factor [75], and 100 U of penicillin/streptomycin per ml.

### Expression plasmids

Plasmid pp89UC codes for the MCMV IE1 protein pp89, carrying the insert of plasmid pIE 100/1 [2] in the pUC19 vector. IE1 expression is under control of its native MCMV major immediate early enhancer/promoter. Plasmid pEYFP-C2 was produced by transferring the EYFP ORF from pEYFP-C1 (BD Biosciences Clonetech) into the pEGFP-C2 vector (BD Biosciences Clonetech), replacing the EGFP ORF using endonucleases AgeI and BsrGI. The expression plasmid pTNF-gLuc was produced by replacing the MCMV enhancer/promoter in the plasmid pmCherryP2AGLucKanR [76] with the TNFα enhancer/promoter (−670 to +1). To do so the TNF enhancer/promoter was synthesised by MWG/Operon including restriction sites for KpnI and BglII, used for the cloning procedure.

### Lipofection

To transfect cultured cells with expression plasmids we used the Lipofect LTX reagent (Invitrogen). IE1 or TNF-reporter plasmids were mixed with the firefly vector pGL3 (Promega) for internal control of transfection efficiency. To ensure that all cultures were exposed to the same amounts of DNA during transfection, mixtures of plasmids were adjusted to the same total amount of DNA within one experiment with the cloning vector pCR3.1 (Invitrogen). For transfection cell line specific protocols provided by Invitrogen were used (detailed protocols available at: www.invitrogen.com/transfections) and adjusted to the respective culture size as described in the NIH3T3 specific protocol.

### Luciferase reporter assays

*Gaussia* luciferase assays were carried out as described previously [76], with the exception that plain DMEM medium was used to produce coelenterazine working solution. Substrate (50 µl) was mixed with 50 µl culture supernatant and measured with a POLARstar plate reader (BMG Labtech, UK). To measure firefly luciferase activity we used the Luciferase Assay System (Promega) as described in the manual. In short, cells were lysed for 15 min on a rocking platform and 15 µl lysate were subsequently transferred into a black/white plate and mixed with 30 µl substrate for measuring luminescence in the POLARstar plate reader. To stimulate luciferase expression, cells were washed 1× in growth medium and then stimulated with LPS (10 or 100 ng/ml in growth medium, as indicated) for 4 h before reporter gene activity in the culture supernatant was measured.

### Characterization of BMMΦ by flow cytometry

Maturation of BMMΦ cells was tested by flow cytometry analysis for the expression of murine proteins specific for mature macrophages. Analyses were performed for F4/80 (Caltag Laboratories, UK) and CD11b (eBiosciences, UK) using a FACScan or FACSCalibur instrument.

### BMMΦ infection

Cells were infected with the different viruses at an MOI of 1, unless specified otherwise. After 1 h of adsorption, cells were washed in PBS and incubated in fresh DMEM:F12 supplemented with 10% FCS, 10% L929, and 100 U of penicillin/streptomycin per ml.

### Mouse infections

8 weeks-old male BALB/c and C57BL/6 mice were obtained from Charles Rivers Lab (Barcelona, Spain and Edinburgh, UK, respectively), C57/Bl6 TNF^−/−^ mice were obtained from B&K Universal (UK). Experiments with C57/Bl6 TNFRp55^−/−^ mice [77] were conducted in the University of Rijeka. Animals were housed at the animal facilities (University of Barcelona, University of Rijeka or Edinburgh University) under pathogen-free conditions. Mice were intraperitoneally inoculated with 3×10^5^ or 2–3×10^6^ PFU of tissue culture-derived MCMV recombinants. At designated times mice were sacrificed and spleen, liver, kidneys, heart and lungs were removed, weighted and harvested as a 10% (wt/v) homogenate. Part of the tissue homogenate was sonicated and viral titres were determined on MEF by standard plaque assay. When infectious virus could not be detected in a particular organ, a titre corresponding to the limit of detection of the assay was assigned to that particular organ in order to calculate the median values.

For *in vivo* analysis of TNFα transcription levels, female BALB/c mice were immune depleted and infected essentially as described in greater detail previously [78]. In brief, hematoablative conditioning of 8- to 9-week-old female mice was achieved by total-body γ-irradiation with a single dose of 6.5 Gy. Intraplantar infection at the left hind footpad was performed ∼2 h later with 10^5^ PFU of either BAC-cloned virus MCMV wild-type [72] or MCMVdie1 [11].

### TNFα levels

Cytokine levels were determined from cell culture supernatants by flow cytometry (BD Cytometric Bead Array, BD Biosciences) and from tissue homogenates by ELISA (mouse TNF-α/TNFSF1A DuoSet ELISA Development kit, R&D Systems Europe Ltd.) following manufacturer's instructions. TNFα concentration was determined by reading the absorbance at 450 nm in a POLARstar OPTIMA Multifunction Microplate Reader (BMG LabTech, UK).

### Immunofluorescence

50 µl of suspension containing 10^4^ cells were added onto each dot of a Teflon coated microdot slide. Cells were incubated at 37°C for 24 h. After infection and treatment, cells were fixed with 4% paraformaldehyde for 10 min and permeabilized in 0.5% Triton X-100 for 3 min. After blocking for 1 h with 20% FCS, cells were stained with primary antibody Croma103 (provided by S. Jonjic) and TNFα (Santa Cruz, SC-1351) and secondary antibody Alexa Fluor 488 rabbit anti-mouse IgG and Alexa FluorAR 594 donkey anti-goat IgG (both from Invitrogen, CA), respectively.

### Nuclear translocation assay

RAW G9 cells (5×10^3^ per well) were seeded in glass bottom optical 384 well plates. After 24 h cells were stimulated with 10 ng/ml LPS or infected with virus in 20 µl. Subsequently cells were fixed with 4% PFA for 30 min (RT), quenched in 50 mM NH_4_Cl solution for 5 min and permeabilised with 0.5% Triton X-100 for 3 min. Cell nuclei were counterstained with DAPI. For analysis pictures were taken on an OPERA system (PerkinElmer, USA) with 40× magnification and for GFP nuclear translocation assay the corresponding standard script of the Acapella (v2.3) analysis software was used.

### Total RNA extraction from BMMΦ

RNA from BMMΦ cells in 6-well plates was extracted by adding 200 µl Trizol reagent (Invitrogen, CA) and incubating for 5 min. Samples were then transferred to a 1.5 ml microfuge tube and 40 µl chloroform were added. Samples were incubated at room temperature for 15 min before being centrifuged (13,000 rpm, 4°C, 5 min). The upper aqueous layer was removed and 0.1 volumes 3 M NaOAc, 2.5 volumes EtOH were added. Samples were incubated (−20°C, 60 min) and then centrifuged (13,000 rpm, 4°C, 30 min). Supernatant was removed and the pellets washed in 200 µl 70% (v/v) ethanol and centrifuged as before. RNA pellets were then resuspended in 50 µl RNAse-free H2O. RNA quantity and quality was assayed by measuring the A260 and the A260/A280 ratio respectively using a Nanodrop ND-1000 (Nanodrop Technologies, DE).

### Isolation of total RNA from liver tissue

RNA was isolated as described in detail previously [10] from whole livers shock-frozen in liquid nitrogen.

### qRT-PCR

For each sample, 2× Taqman PCR mix (Applied Biosystems; CA) was mixed with 40 U of Superscript III (Invitrogen, CA). 4 µl total RNA was then added and each sample split into two reactions. A Taqman primer/probe set (Applied Biosystems, CA) for the gene of interest was then added to one reaction at the recommended concentration while a Taqman primer/probe set for GAPDH mRNA was added to the other reaction. Samples were then run on a MX1000P quantitative PCR thermal cycler (Stratagene, CA). Samples were first heated to 50°C for 30 minutes then heated to 95°C for 10 minutes. Samples were then subjected to 40 cycles under Taqman standard conditions. Stratagene MXPro software was used to analyse the data.

### Quantification of transcripts

Quantification of β-actin and E1 transcripts was performed with homemade primers and probes as described previously, yielding amplicons of 88 bp [10] and 200 bp [79], respectively.

Quantification of IFNβ, IL10 and TNFα transcripts was done with custom-made TaqMan Gene Expression Assays.

- **IFN**β **transcripts.** TaqMan Gene Expression Assay (catalog no. Mm00439546-s1; Applied Biosystems). Amplicon length: 61 bp.
- **IL10 transcripts.** TaqMan Gene Expression Assay (catalog no. Mm00439614-m1; Applied Biosystems). Amplicon length: 79 bp.
- **TNFα transcripts.** TaqMan Gene Expression Assay (catalog no. Mm00443258-m1; Applied Biosystems). Amplicon length: 81 bp.

Reactions were performed in a total volume of 20 µl, including 4 µl of 5 X QIAGEN OneStep RT-PCR buffer, 1 µl of QIAGEN OneStep RT-PCR enzyme mix, 668 µM of each dNTP, 1.5 mM additional MgCl_2_, 0.132 µM 5-carboxy-X-rhodamine as passive reference, and 0.6 µM of each β-actin primer and 0.26 µM of β-actin probe, or 1 µl of the corresponding 20× TaqMan Gene Expression Assay Mix (in the case of IFNβ, IL10, and TNFα). Reverse transcription was performed at 50°C for 30 min. The cycle protocol for cDNA amplification started with an activation step at 95°C for 15 min, followed by 40 cycles of denaturation for 15 sec at 95°C and a combined primer annealing/extension step for 1 min at 60°C during which data collection was performed. The efficiencies of the RT-PCRs were >90% throughout. Relative quantifications were made with the comparative C_T_ (cycle threshold) method as described previously [10], [79].

### Quantification of viral genomes

Number of viral genomes was determined by measuring copy numbers of the viral M115 gene as described previously [41].

### Quantification of infected cells in liver tissue

Immunohistochemical staining of major capsid protein (MCP; M86) present within inclusion bodies in the nuclei of infected cells was performed in liver tissue sections as described previously [10].

### Western blot

Whole lysate was extracted from non-infected and infected-BMMΦ using Beadlyte® Cell Signalling Lysis Buffer (Milipore, UK), following manufacturer's instructions. BMMΦs were cultured for 7 days in 6-well plates at a seeding density of 10^6^ cells/well. Prior to infection, cells were serum starved for 24 h. After infection cells were washed with ice-cold TBS and lysed in lysis buffer, containing Complete Miniprotease Inhibitor (Roche, UK) and Phosphatase Inhibitor Cocktail I and II (both from Sigma, UK). Protein concentration was determined using the MicroBCA protein assay (Pierce, UK), following manufacturer's instructions. Equal amounts of protein were mixed with 2× Laemmli Sample Buffer, containing 10% of DTT and loaded onto a 10% SDS-PAGE gels. Proteins were transferred to a PVDF membrane which was then probed with anti-phosphor-p38, anti p38, anti-phosphor-JNK, anti JNK (Cell Signaling, UK), and anti IκBα (Sigma, UK) and incubated with HRP-conjugated anti mouse or rabbit IgG (Cell Signalling, UK). Proteins were detected with ECL Plus Western Blotting Detection Reagents (Amersham Biosciences, UK) using VersaDoc imaging system 4000. Densitometric analysis of the blots was performed by Quantity One software 4.5.0. For pp38 MAPK and IE1 expression plasmid experiments, RAW 264.7 cells were washed with PBS and resuspended in whole-cell lysis buffer (50 mM Tris-HCl, pH 7.5, 100 mM NaCl, 1% NP40, protease inhibitors, and phosphatase inhibitors), and cell lysates were centrifuged at 4°C for 10 min and the collected supernatants were stored at −20°C. Protein concentration was measured by Pierce BCA assay (Thermo Scientific). For Western blotting, proteins were separated by 10% SDS-PAGE, transferred to Immobilon-FL membranes (Millipore), and probed with rabbit anti-p38α (Santa Cruz, sc-27578, 1∶2000), mouse anti-pp38 MAPK (Cell signalling, 9216L, 1∶1000), and rabbit anti-β-actin (Cell Signalling, 4970, 1∶2500) diluted in PBST (0.1% Tween20). For secondary anti-rabbit IR-680 (Invitrogen, A21109, 1∶10,000), IR-800 anti-mouse (Thermo Fisher Scientific, 35571, 1∶10,000), antibodies were diluted in PBST (0.1% Tween20). For visualization, the Odyssey protocol (LI-COR) was followed. The fluorescence was quantified using ImageJ (ver. 1.45s).

### Statistical analysis

Statistical analysis was performed in MATLAB (2007, The MathWorks, Inc). Viral titres and cytokine levels from *in vivo* experiments were compared by using Mann-Whitney U test. Analysis from *in vitro* experiments was compared by Student's t-test.

### Normalisation procedure

For data normalisation, values of respective control (e.g. firefly activity) for each sample were expressed relative to the average of the control in the respective experiment. This normalisation factor was then used to correct the corresponding measured value in the assay (e.g. gLuc activity).

### Gene accession numbers

(Entrez Gene ID) TNFa (Tnf, 21926), TNFR (Tnfrsf1a, 21937), p38 (Mapk14, 26416), NFκB-p65 (Rela, 19697), JNK (Mapk8, 26419), Ifnb1 (15977), IL10 (16153), CD11b (Itgam, 16409), F4/80 (Emr1, 13733), Ifng (15978), MKP-1 (Dusp1, 19252), IL12A (3592)

## Supporting Information

Figure S1 **Characterization of BMMΦ by flow cytometry.** Maturation of day 7 BMMΦ assessed by staining for the specific expression of murine MΦ cell surface proteins F4/80 and CD11b. **A.** FACS dot blot showing the gating forward scatter (FSC) and side scatter (SSC). (B) This panel shows the population of F4/80^+^CD11b^+^ MΦ (93.1%). Histograms for F4/80 (C) and CD11b (D) staining are also shown.
(TIF)

Figure S2 **Positive activation of MΦ after LPS stimulation.** As a control for normal activation of cells, RAW264.7 macrophages were stimulated with LPS for 6 h. Cells were then fixed with 4% paraformaldehyde and staining was performed for TNFα. Cytokine production was compared to mocked-stimulated cells. DNA was counterstained with DAPI.
(TIF)

Figure S3 **Correlation between TNFα levels and infectious virus in heart and kidney after 4 days of MCMVrev infection.** Pearson's correlation coefficient shows a significant correlation between the levels of cytokine produced and PFU per gram of tissue in kidneys (A) and heart (B) from MCMVrev-infected BALB/c mice for 4 days.
(TIFF)

Figure S4 **Infection with MCMV and MCMVdie1 suppresses NFκB activation and translocation to the nucleus.** (A) RAW G9 cells, stably expressing a NFκB(p65)-GFP fusion protein, were infected or LPS treated and 45 min after the first contact with virions cells were fixed in 4% PFA, counterstained with DAPI and GFP translocation was monitored with an OPERA system (PerkinElmer). (B) Quantification of cytoplasmic and nuclear GFP fluorescence in treated cells. OPERA Acapella analysis software was used to quantify fluorescence in treated cells in 3 different snapshots of 4 wells per respective treatment. A standard fluorescence translocation script from the Acapella software (“NFkB Cytoplasm to Nuclei Translocation Assay”) with standard settings was used for quantification of nuclear NFκB translocation. Bars represent averages of normalised fluorescence with SE.
(TIF)

Figure S5 **Viral replication in organs of TNF^−/−^ and TNFR^−/−^ mice.** Organs from infected C57B/6, TNFα^−/−^ (A) or Balb/c TNFRp55^−/−^ (B) mice (2×10^6^ PFU i.p.) were harvested at 4 dpi and homogenated for analysis with standard plaque assay (MCMV = grey circles; MCMVdie1 = open circles). Titres were normalised per sample weight, black lines indicate median values and dashed line represents limit of detection.
(TIFF)

Figure S6 **Viral replication in organs of Bl6 mice treated with 100 ug of anti-TNFα antibody (IP) at day 0 and day 2 p.i.** Organs from infected mice (3×10^5^ PFU i.p.) were harvested at 4 dpi and homogenated for analysis with standard plaque assay. Black lines indicate median values and dashed line represents limit of detection.
(TIF)

Figure S7 **Quantification of LiCor western blot images for detection of p38/pp38 and pJNK.** Average of quantification (n = 3) of one representative experiment is shown, error bars represent SE of quantification. MCMVdie1 is abbreviated as die1 in this figure. (A) Quantification of pp38/p38 levels and normalised signal intensity in plasmid transfected RAW cells after LPS stimulation. (B) Quantification of pp38/p38 and pJNK levels and normalised signal intensity in infected RAW cells at 10 hpi after LPS stimulation. (C) Quantification of pp38/p38 levels and normalised signal intensity infected RAW cells at 4 hpi after LPS stimulation. (D) Densitometric quantification of IκBα, pp38 and pJNK levels at 10 hpi.
(TIF)

Figure S8 **Characterisation of fluorochrome conjugated primary anti-TNF antibody by flow cytometric analysis.** BMMΦs were stimulated with indicated concentration of LPS and stained with anti-TNF antibody or isotype control antibody. Fluorescence was analysed using a FACScan instrument.
(TIF)
