# Supplementary figures and images for: Ablation of the Regulatory IE1 Protein of Murine Cytomegalovirus Alters In Vivo Pro-inflammatory TNF-alpha Production during Acute Infection
Source: PLoS Pathog. 2012 Aug 30;8(8):e1002901. doi: 10.1371/journal.ppat.1002901 (PMC3431344; doi:10.1371/journal.ppat.1002901)

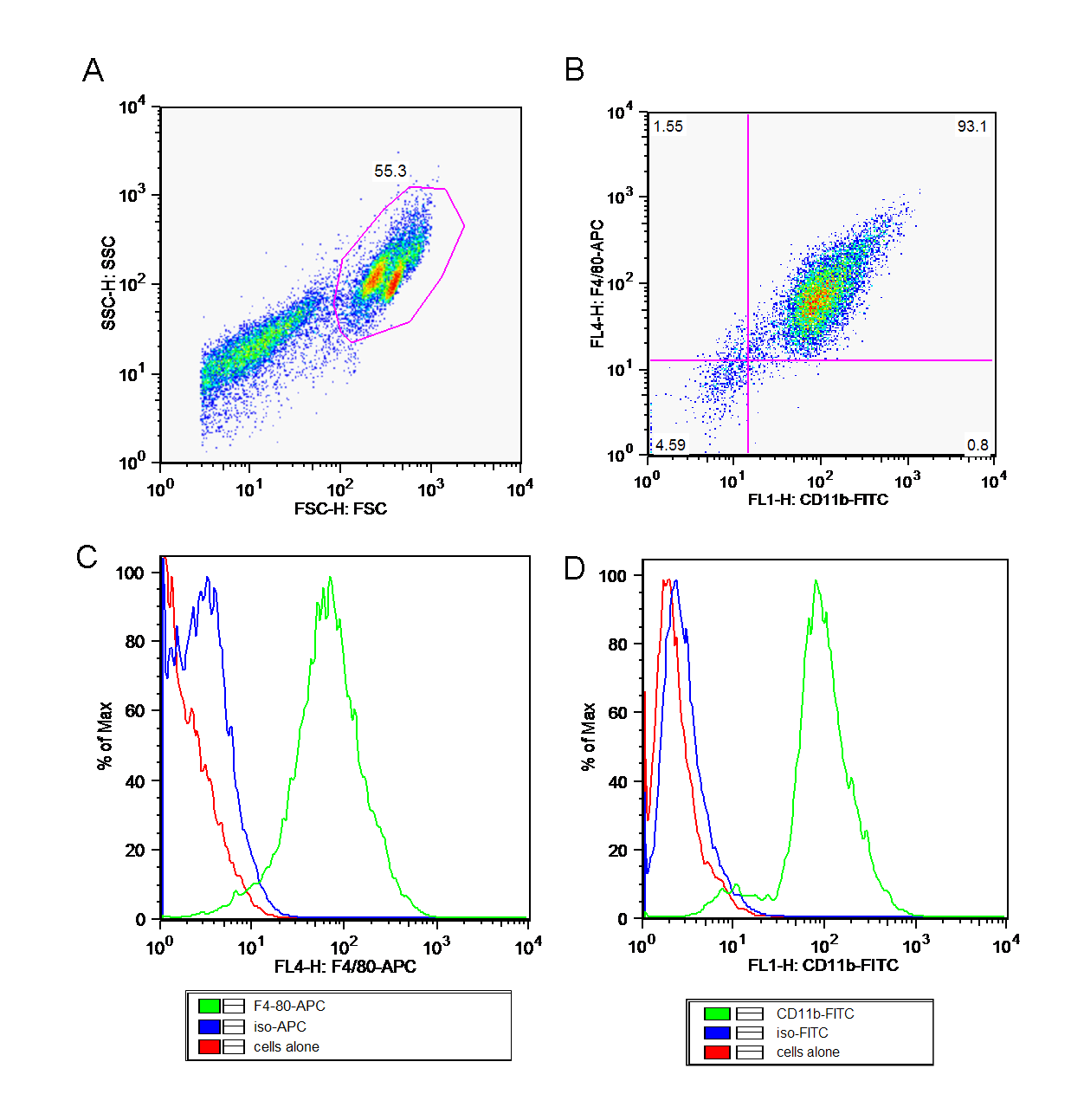

Supplement: Figure S1 — Characterization of BMMΦ by flow cytometry. Maturation of day 7 BMMΦ assessed by staining for the specific expression of murine MΦ cell surface proteins F4/80 and CD11b. A. FACS dot blot showing the gating forward scatter (FSC) and side scatter (SSC). (B) This panel shows the population of F4/80+CD11b+ MΦ (93.1%). Histograms for F4/80 (C) and CD11b (D) staining are also shown. (TIF) [file ppat.1002901.s001.tif]

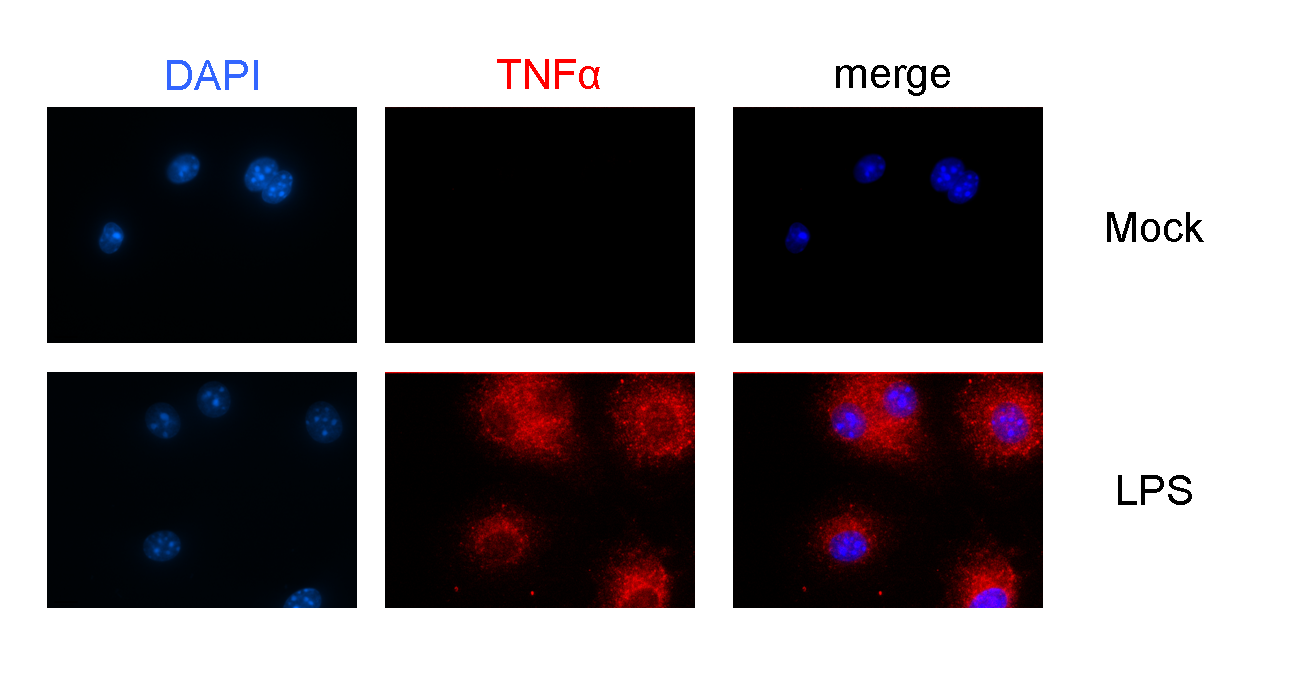

Supplement: Figure S2 — Positive activation of MΦ after LPS stimulation. As a control for normal activation of cells, RAW264.7 macrophages were stimulated with LPS for 6 h. Cells were then fixed with 4% paraformaldehyde and staining was performed for TNFα. Cytokine production was compared to mocked-stimulated cells. DNA was counterstained with DAPI. (TIF) [file ppat.1002901.s002.tif]

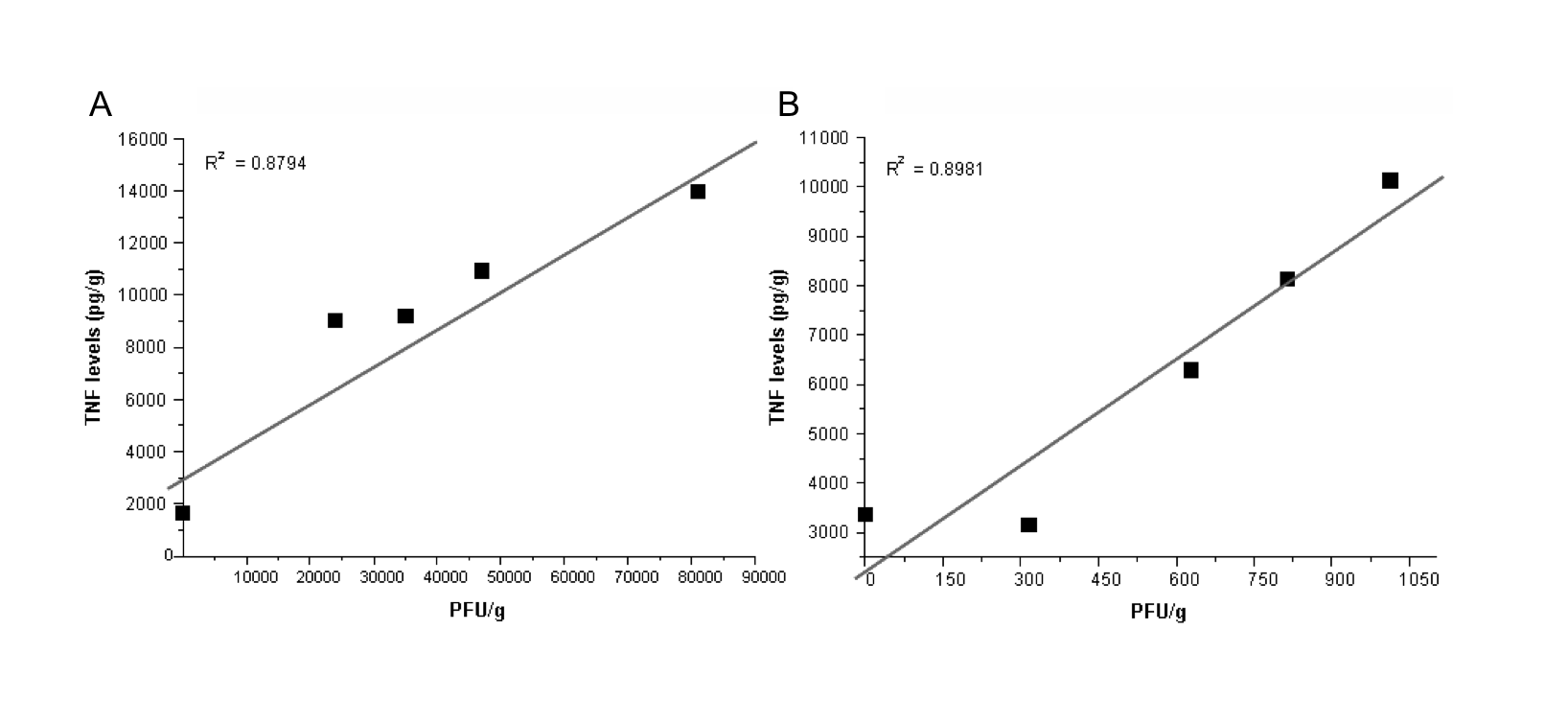

Supplement: Figure S3 — Correlation between TNFα levels and infectious virus in heart and kidney after 4 days of MCMVrev infection. Pearson's correlation coefficient shows a significant correlation between the levels of cytokine produced and PFU per gram of tissue in kidneys (A) and heart (B) from MCMVrev-infected BALB/c mice for 4 days. (TIFF) [file ppat.1002901.s003.tiff]

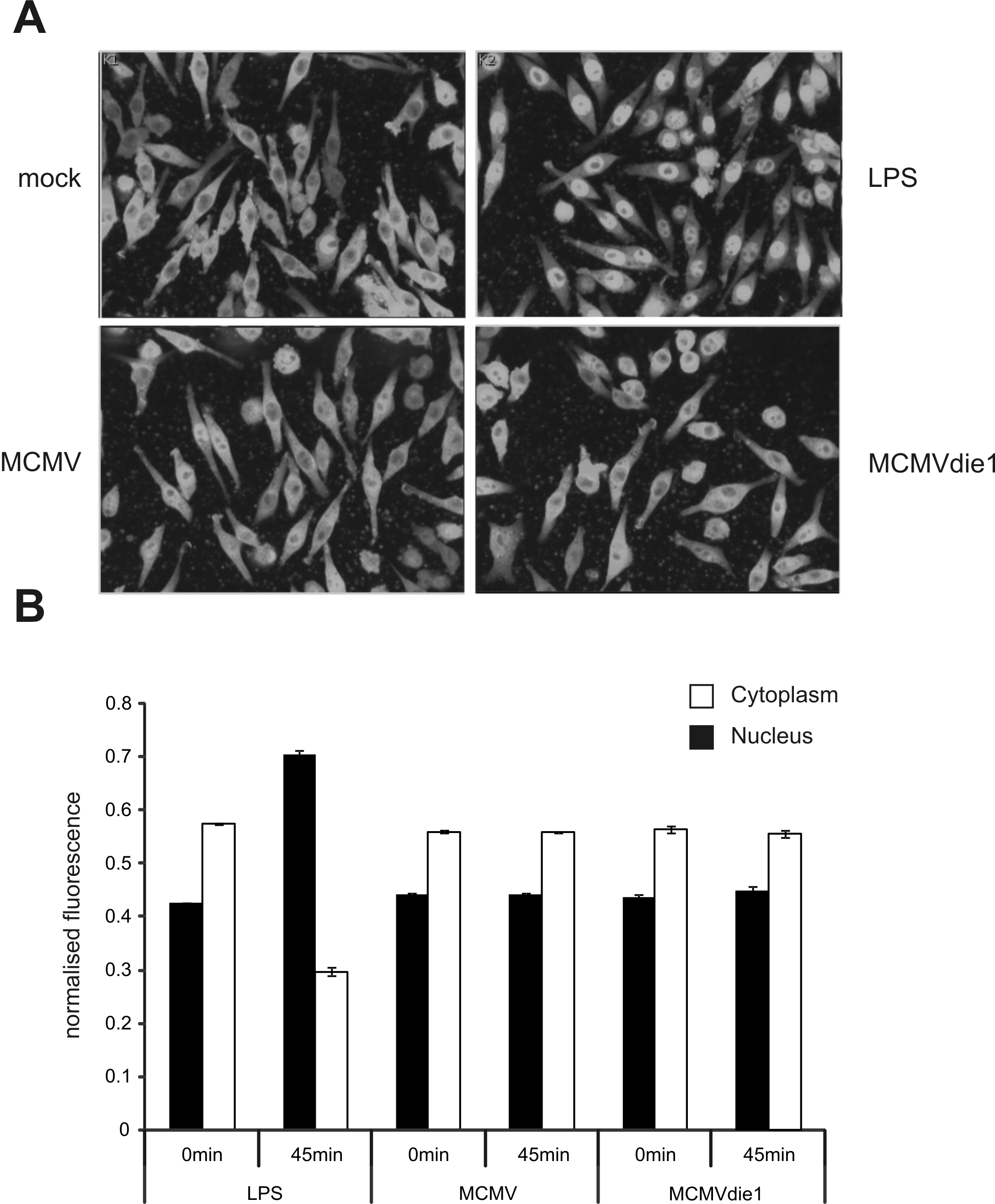

Supplement: Figure S4 — Infection with MCMV and MCMVdie1 suppresses NFκB activation and translocation to the nucleus. (A) RAW G9 cells, stably expressing a NFκB(p65)-GFP fusion protein, were infected or LPS treated and 45 min after the first contact with virions cells were fixed in 4% PFA, counterstained with DAPI and GFP translocation was monitored with an OPERA system (PerkinElmer). (B) Quantification of cytoplasmic and nuclear GFP fluorescence in treated cells. OPERA Acapella analysis software was used to quantify fluorescence in treated cells in 3 different snapshots of 4 wells per respective treatment. A standard fluorescence translocation script from the Acapella software (“NFkB Cytoplasm to Nuclei Translocation Assay”) with standard settings was used for quantification of nuclear NFκB translocation. Bars represent averages of normalised fluorescence with SE. (TIF) [file ppat.1002901.s004.tif]

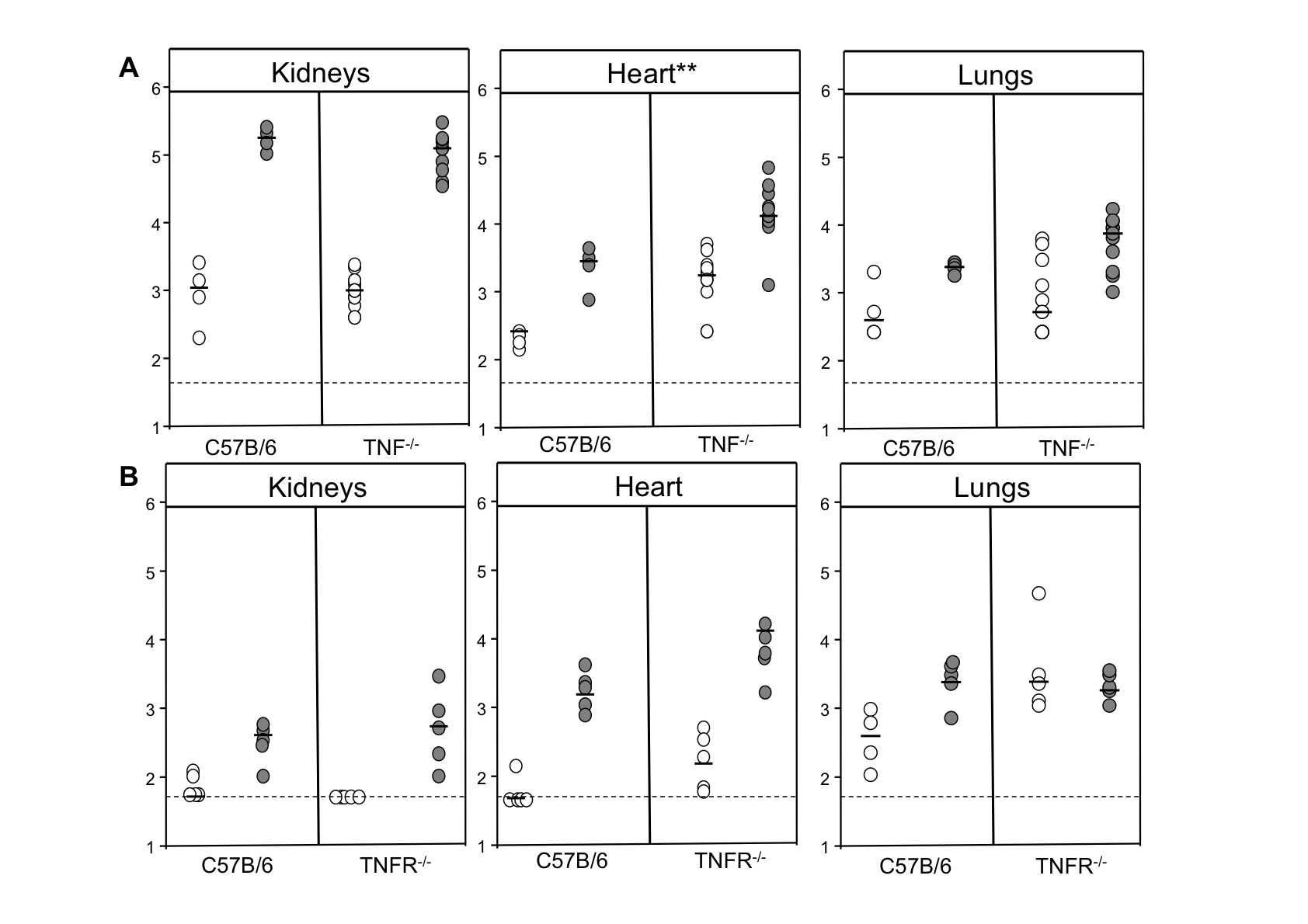

Supplement: Figure S5 — Viral replication in organs of TNF−/− and TNFR−/− mice. Organs from infected C57B/6, TNFα−/− (A) or Balb/c TNFRp55−/− (B) mice (2×10^6^ PFU i.p.) were harvested at 4 dpi and homogenated for analysis with standard plaque assay (MCMV = grey circles; MCMVdie1 = open circles). Titres were normalised per sample weight, black lines indicate median values and dashed line represents limit of detection. (TIFF) [file ppat.1002901.s005.tiff]

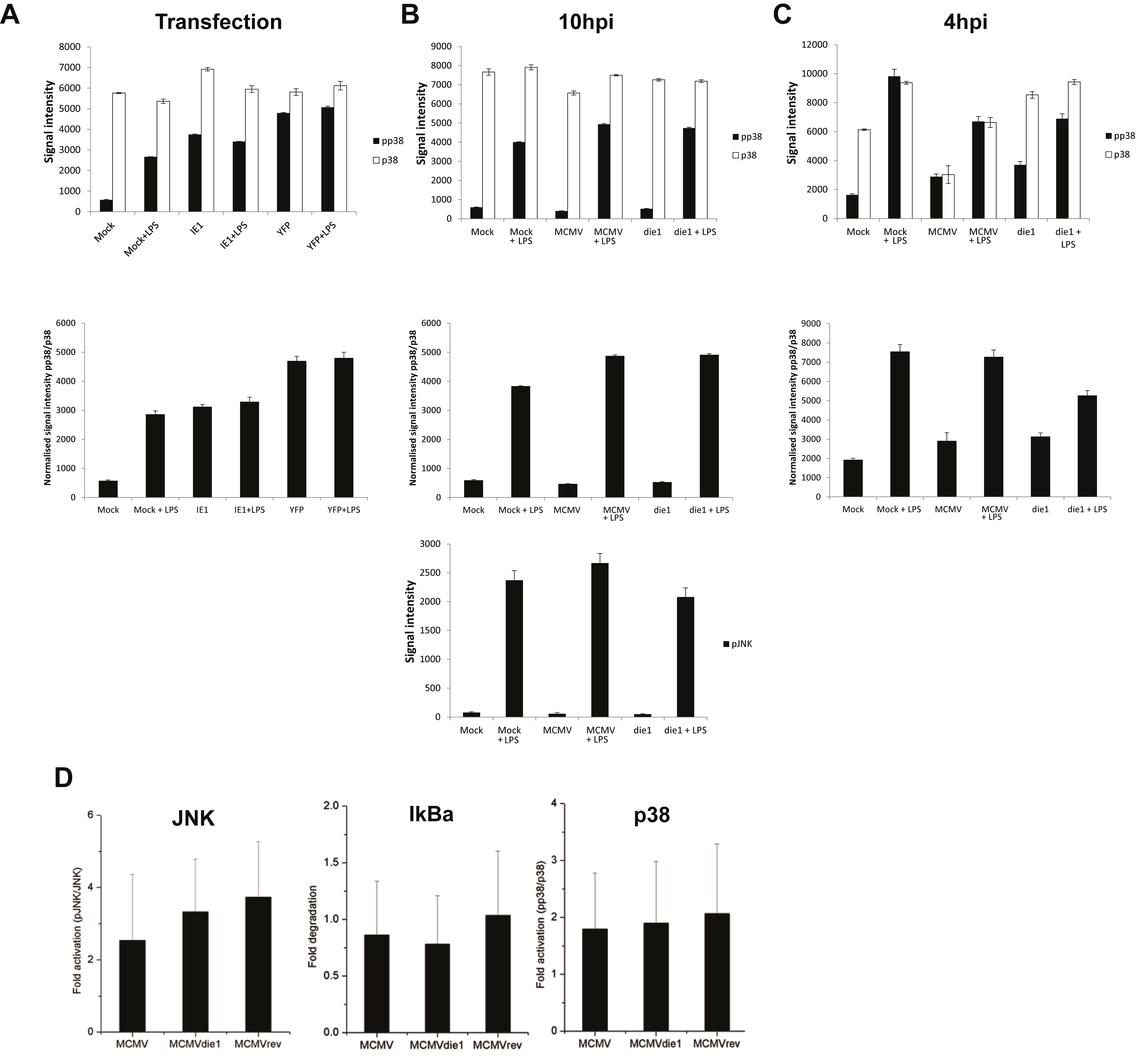

Supplement: Figure S7 — Quantification of LiCor western blot images for detection of p38/pp38 and pJNK. Average of quantification (n = 3) of one representative experiment is shown, error bars represent SE of quantification. MCMVdie1 is abbreviated as die1 in this figure. (A) Quantification of pp38/p38 levels and normalised signal intensity in plasmid transfected RAW cells after LPS stimulation. (B) Quantification of pp38/p38 and pJNK levels and normalised signal intensity in infected RAW cells at 10 hpi after LPS stimulation. (C) Quantification of pp38/p38 levels and normalised signal intensity infected RAW cells at 4 hpi after LPS stimulation. (D) Densitometric quantification of IκBα, pp38 and pJNK levels at 10 hpi. (TIF) [file ppat.1002901.s007.tif]

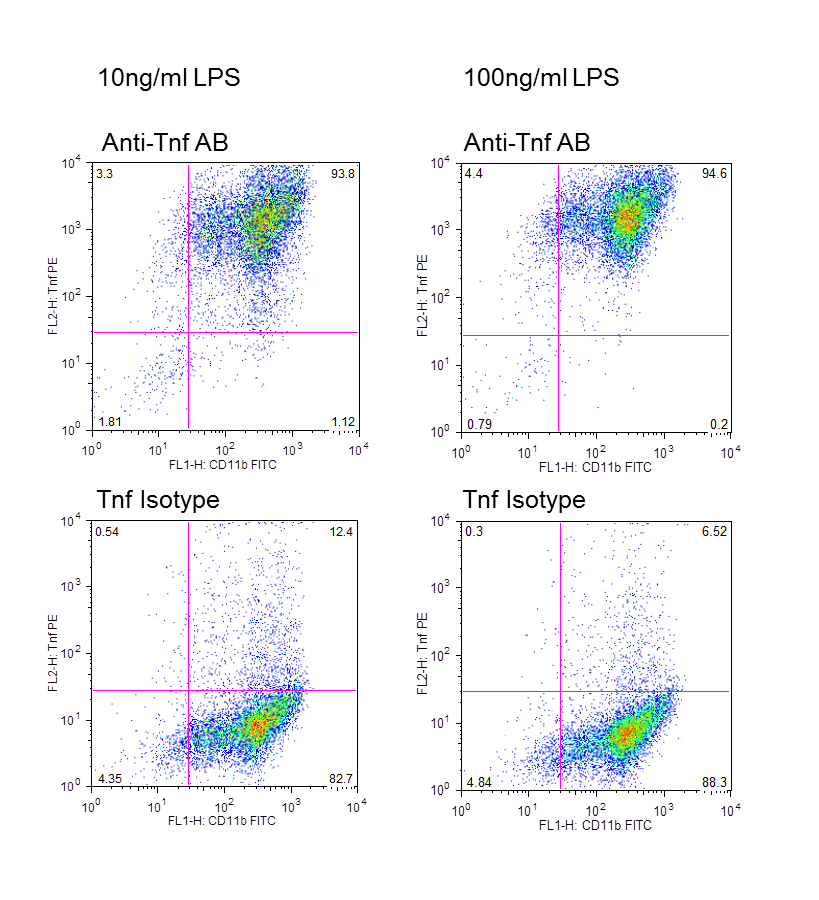

Supplement: Figure S8 — Characterisation of fluorochrome conjugated primary anti-TNF antibody by flow cytometric analysis. BMMΦs were stimulated with indicated concentration of LPS and stained with anti-TNF antibody or isotype control antibody. Fluorescence was analysed using a FACScan instrument. (TIF) [file ppat.1002901.s008.tif]
